# A novel Hankel norm approximation-based AGC for a hydro-dominated power system

**DOI:** 10.1038/s41598-026-35235-9

**Published:** 2026-01-16

**Authors:** Sadaf Naqvi, Ibraheem, Gulshan Sharma, Rajesh Kumar, Sachin Sharma

**Affiliations:** 1 Department of Electrical Engineering, Jamia Millia Islamia, New Delhi, 110025 India; 2 Department of Electrical Engineering, Netaji Subhas University of Technology, New Delhi, 110078 India; 3 Department of Electrical & Electronic Engineering Technology, University of Johannesburg, Johannesburg, 2006 South Africa; 4 Department of Human Anatomy and Physiology, Faculty of Health Sciences, University of Johannesburg, Johannesburg, 2094 South Africa; 5 Department of Electrical Engineering, Malaviya National Institute of Technology, Jaipur, 302017 India; 6 Manipal Institute of Technology, Manipal Academy of Higher Education, Manipal, 576104 Karnataka India

**Keywords:** Automatic generation control (AGC), Model order reduction (MOR) techniques, Hankel norm approximation (HNA), Truncation method, Multivariable system, Modeling & simulation, Hydro turbine, Electrical and electronic engineering, Energy science and technology

## Abstract

Analyzing power system disturbances for automatic generation control (AGC) requires solving a large set of differential equations, which remains computationally demanding even in linearized form and hence limits the practical implementation of most control strategies. In addition, the presence of time constants and delays further complicates the modeling by influencing the response of generators, governors, and control mechanisms especially for hydro dominated power systems. To address these challenges, Model Order Reduction (MOR) techniques play an important role in solving these issues for higher order and complex systems. This paper applies the Hankel norm approximation (HNA) method to develop reduced-order models for AGC in a hydro-dominated power system. An eleventh-order model is reduced to seventh, eighth and ninth-order representations. Stability margins of these reduce order models are evaluated through eigenvalue analysis, and the dynamic responses of the reduced order models are compared against the original model. Results demonstrate that the reduced order models preserve the essential dynamics while substantially lowering computational effort in AGC. To further assess the effectiveness of HNA, a Truncation-based reduction approach is also applied, and comparative results are offered to show the benefits of the proposed work.

**Supplementary Information:**

The online version contains supplementary material available at 10.1038/s41598-026-35235-9.

## Introduction

Modelling and simulation play a crucial role in analyzing power system problems and models. The modelling process involves solving numerous differential equations, and as the complexity of a system increases, its model becomes larger and more intricate. These complex models are not only time-consuming to solve but also challenging to interpret. To enhance system understanding and reduce computational effort, simplified models are necessary. Over the past few decades, extensive research has been conducted on reducing system order. Model Order Reduction (MOR) techniques have emerged as essential tools for minimizing system complexity, as discussed in^1^. The application of one such technique, Singular Perturbation, to power electronics devices was explored in^2^, demonstrating a significant reduction in simulation time. The effectiveness of MOR techniques has been validated across various models, as reported in^3–6^.

A wide range of MOR techniques is available in the literature and has been implemented in different complex systems. These methods not only improve computational efficiency but also optimize memory usage. For single-input, single-output (SISO) systems, applying MOR techniques is relatively straightforward. However, reducing the complexity of multi-input, multi-output (MIMO) systems present additional challenges^7^.

Among the most widely used time-domain reduction methods are the Balanced Truncation method and the Optimal Hankel Norm Approximation (HNA) method. Both techniques share a fundamental connection with Singular Value Decomposition (SVD), ensuring system stability while enabling the estimation of error between the frequency responses of the full-order and reduced-order models. Virappaniyan et al.^8^ utilized the HNA matrix for the stability analysis of nonlinear dynamic systems. Additionally, an optimal HNA algorithm for multivariable systems was introduced by Yang & Yeh in^9^ and later extended in^10,11^. A more efficient approach for reducing large-scale systems using HNA was further developed by Peter Benner et al. in^12^.

The application of model order reduction to discrete systems was explored in^13^. Automatic Generation Control (AGC) remains a complex and evolving challenge in power systems, with various control strategies available for AGC studies^14,15^. However, hydro power is gaining momentum as a preferred energy source due to its ability to generate electricity without harmful environmental emissions^16^. In^17^, AGC studies on hydro systems were conducted with an emphasis on optimization techniques incorporating Flexible AC Transmission Systems (FACTS). Similarly, in^18^, a fuzzy logic-based approach was integrated with optimization methods to address AGC challenges in hydro systems. A novel concept of interlinked hydro systems in collaborating optimization approach with linear quadratic regulator (LQR) was discussed in^19^. However, it has been observed that hydro power systems are complex with higher order of the system model and hence poses a challenge for researchers, necessitating the development of feasible and practical solutions for AGC implementation. HNA has the potential to effectively reduce power system models while maintaining system dynamic performance and response integrity^20–22^.

### Research gap and motivation

Although a variety of Model Order Reduction (MOR) techniques have been successfully applied to simplify dynamic models, most existing studies are limited to thermal-dominated or single-input, single-output (SISO) systems. The application of advanced MOR methods to complex multi-input, multi-output (MIMO) models, particularly in hydro-dominated systems, remains underexplored. Hydro-based systems present unique modeling challenges due to large time constants, nonlinearities, and inherent delays, making it difficult for conventional approaches such as truncation or singular perturbation to preserve essential dynamics. At the same time, the growing integration of renewable energy sources (RES) has increased the need for efficient and accurate models. The RES integration introduces variability, intermittency, and complex interactions that demand simpler, accurate and reliable control strategies. Hydro power plants, being one of the most controllable and stable renewable resources, play a crucial role in supporting these days power system operations by providing inertia, frequency regulation, and storage-like characteristics. However, the computational burden of simulating higher order models of power system having hydro turbines poses a major limitation to real-time implementation. This creates a clear research gap and motivates the need to develop and validate advanced MOR approaches, such as HNA, that can significantly reduce model complexity while retaining system dynamics. In addition, it is quite difficult to obtain the information regarding all states and hence reduced-order models will not only enhance computational efficiency but also facilitate practical controller design and secure reliable operation of higher order and complex power system.

### Contribution and outline of the article

- This paper aims to address the AGC problem by applying the HNA method. The research focuses on the mathematical modelling and implementation of HNA in a two-area interlinked power system with hydro-based generation.
- The study presents results for the original system model along with its reduced seventh, eighth and nineth order versions, highlighting the advantages of the proposed approach. The accuracy of these reduced models is validated through eigenvalue analysis and system response evaluations for AGC across different model orders.
- The study demonstrates that the application of HNA enable the development of simplified models that retain essential AGC dynamics in a two-area system. These reduced models offer a practical balance between accuracy and complexity, making them more efficient for control design, analysis, and real-world implementation.
- In addition, the graphical AGC responses obtained via HNA is also matched with Truncation-based reduction approach and the application results shows the benefits and importance of the proposed research work.

Outline of this paper is as follows: Section II provides a detailed explanation of the HNA method. Section III presents the mathematical model of the original system and derives its reduced-order versions. Section IV applies the Truncation method to the same model, allowing a comparative analysis with HNA. Section V discusses the results and key findings, followed by the conclusions.

## Hankel norm approximation

One of the most popular methods in time domain is Hankel norm approximation method. The method is based on Hankel singular values of the system. In control theory the Hankel Singular Values give the information of energy which is associated with each state of the system. The higher the energy of the states, the higher the possibility of preserving characteristics of the original system. In HNA method energy of each state is determined using Hankel singular values, this is helpful to decide which state is to be preserved in reduced model. The HNA method is explored by many authors for different models as presented in^20,21^. This method is based on Hankel singular values of the system. Hankel singular values show the high energy state of the system. This method also preserves stability. Using the energy states of singular values of the system one can choose the order of the reduced model. “One of the advantages of this technique is that a priori additive approximation error upper and lower bound can be obtained by using full order system’s Hankel singular values^22^”.

An $\:{n}^{th}$ order original state space system can be represented by

1 $$\:\frac{dx\left(t\right)}{dt}=A\:x\left(t\right)+B\:u\left(t\right)$$

2 $$\:y\left(t\right)=C\:x\left(t\right)+D\:u\left(t\right)$$

where $\:(\mathrm{A},\mathrm{B},\mathrm{C},\mathrm{D})$ are the matrices of appropriate dimensions. A reduced model can be obtained using Hankel norm approximation which can be represented by:

3 $$\:\frac{d{x}_{red}\left(t\right)}{dt}={A}_{red}\:x\left(t\right)+{B}_{red}\:u\left(t\right)$$

4 $$\:{y}_{red}\left(t\right)={C}_{red}\:x\left(t\right)+{D}_{red}\:u\left(t\right)$$

The Hankel norm of stable rational transfer function is defined as

5 $$\left\| {G\left( s \right)} \right\| = \lambda _{{max}}^{{ - \frac{1}{2}}} \left( {W_{c} W_{o} } \right)$$

Where $\:{\lambda\:}_{max}$ stands for the largest eigenvalue. It provides a measure of most controllable /observable state. The square roots of the eigenvalues of the matrix product $\:{W}_{c}{W}_{o}$ are the hankel singular values of a stable transfer function G(s). For a balanced realization the matrices A, B,C can be written in the following form

$$\:\stackrel{-}{A}=\left(\begin{array}{cc}{\stackrel{-}{A}}_{11}&\:{\stackrel{-}{A}}_{12}\\\:{\stackrel{-}{A}}_{21}&\:{\stackrel{-}{A}}_{22}\end{array}\right)\:;\:{\stackrel{-}{A}}_{11}\in\:{\mathbb{R}}^{r\times\:r}\:;$$

$$\:\stackrel{-}{B}=\left(\begin{array}{c}{\stackrel{-}{B}}_{1}\\\:{\stackrel{-}{B}}_{2}\end{array}\right),\:\:\:\:{\stackrel{-}{B}}_{1}\in\:{\mathbb{R}}^{r\times\:m},$$

$$\:\stackrel{-}{C}=\left(\begin{array}{cc}{\stackrel{-}{C}}_{1}&\:{\stackrel{-}{C}}_{2}\end{array}\right),\:{\stackrel{-}{C}}_{1}\in\:{\mathbb{R}}^{p\times\:r}\:;$$

Let Hankel singular values be.

$$\:{\sigma\:}_{1}>{\sigma\:}_{2}>\dots\:\dots\:>{\sigma\:}_{r+1}>{\sigma\:}_{r+2}\dots\:..{\sigma\:}_{n}>0$$

$$\:\varSigma\:=\:diag\:({\sigma\:}_{1},{\:\sigma\:}_{2},\dots\:\dots\:,{\sigma\:}_{r},{\sigma\:}_{r+2}\dots\:..{\sigma\:}_{n},\:{\sigma\:}_{r+1})$$

or $$\:\varSigma\:=\:diag\:({\varSigma\:}_{1},\:{\sigma\:}_{r+1})$$.

Then calculate67

8 $$\:\widehat{C}={C}_{1}{\varSigma\:}_{1}+{\sigma\:}_{r+1}U{B}_{1}^{T}\:\:\:$$

9 $$\:\widehat{D}=D+{\sigma\:}_{r+1}U$$

Where $={{\varSigma\:}_{1}}^{2}-{{\sigma\:}_{r+1}}^{2}I\:$and$\:\:U=-{C}_{2}{\left({B}_{2}^{T}\right)}^{+}\:$Where $\:{\left({B}_{2}^{T}\right)}^{+}$denotes the pseudo inverse of $\:{B}_{2}^{T}$.

Then reduced order model is given by

$$\:{A}_{red}={\widehat{A}}_{11},\:{B}_{red}={\widehat{B}}_{1}\:and\:{C}_{red}={\widehat{C}}_{1}$$

## Application to AGC of a hydro dominating power system

**Fig. 1:**
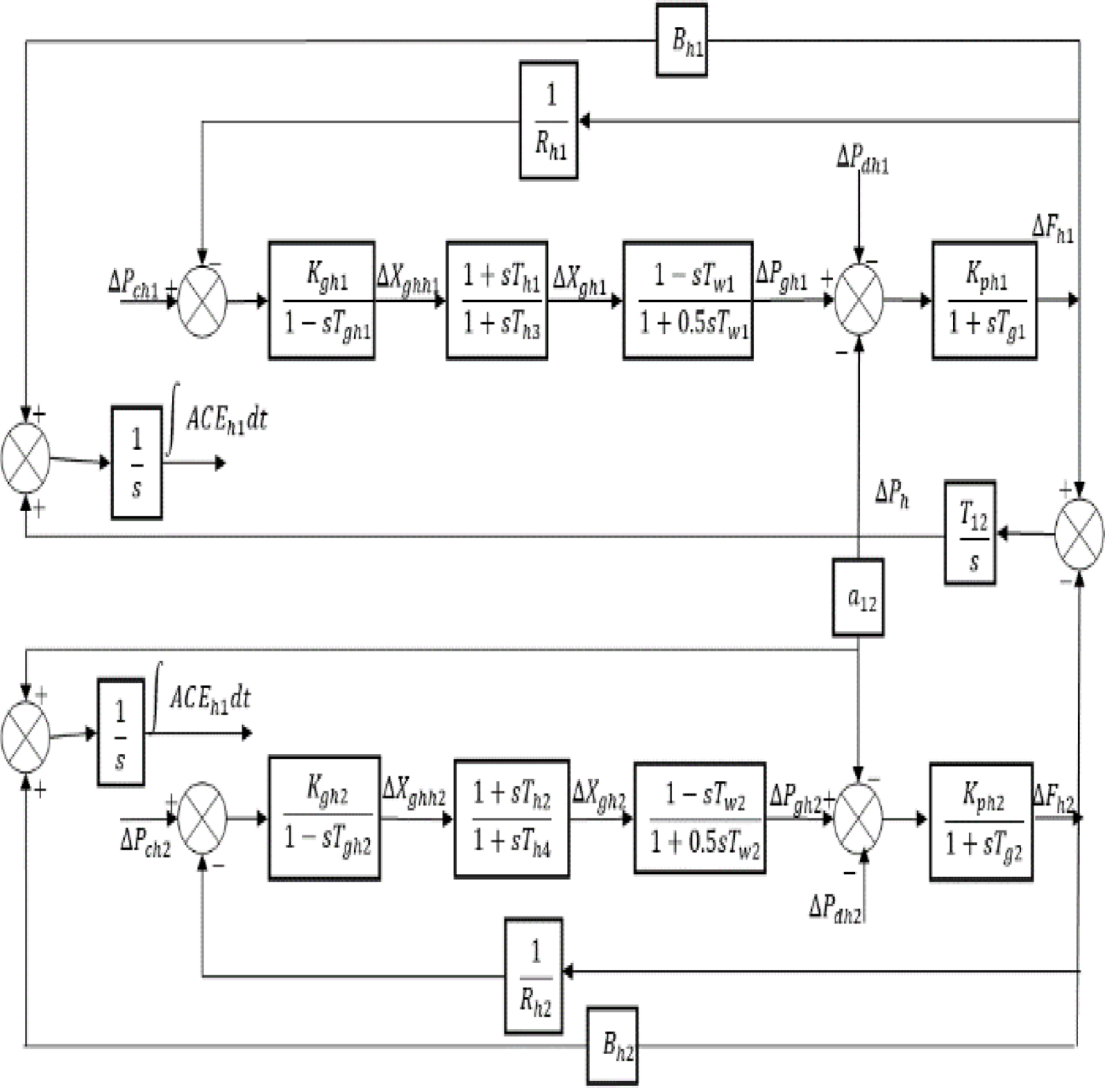
Block diagram model of two-area interlinked power system with hydro turbines.

The block diagram of power system model under consideration with transfer function representation is shown in Fig. 1. It is an interlinked system consisting of plants with same capacity and having hydro turbines generating electrical energy in each area and interlinked via means of AC tie-line. For HNA analysis and reduction of system order, the very first step is to develop the state space model of the power system by solving the differential equations.

The state space equations of power system model can be described as;

10 $$\:\frac{dx}{dt}=Ax+Bu$$

11 $$\:y=Cx+Du$$

The State Vector ‘X’ $\:(11\times\:1),$ Control Vector ‘U’ $\:(2\times\:1)$ and the Disturbance Vector ‘W’ $\:(2\times\:1)$ are:

$$\:X={\left[\begin{array}{ccccccccc}{x}_{1}&\:{x}_{2}&\:{x}_{3}&\:{x}_{4}&\:{x}_{5}&\:{x}_{6}&\:{x}_{7}&\:{x}_{8}&\:{x}_{9}\end{array}\:\:\:{x}_{10}\:\:{x}_{11}\right]}^{T};$$

$$\:U=\left[\begin{array}{c}{u}_{1}\\\:{u}_{2}\end{array}\right];$$

$$\:W=\left[\begin{array}{c}{w}_{1}\\\:{w}_{2}\end{array}\right]$$

The states of the system are:

$$\:{x}_{1}=\varDelta\:{F}_{h1}\:\:\:\:\:\:\:\:\:\:\:\:\:\:{x}_{2}=\varDelta\:P{g}_{h1}\:\:\:\:\:\:\:\:{x}_{3}=\varDelta\:X{g}_{h1}\:\:\:\:\:\:\:\:\:\:\:\:{x}_{4}=\varDelta\:Xg{h}_{h1}\:\:\:\:\:\:\:\:{x}_{5}=\varDelta\:{F}_{h2}$$

$$\:{x}_{6}=\:\varDelta\:P{g}_{h2}\:\:{x}_{7}=\varDelta\:X{g}_{h2}\:{x}_{8}=\varDelta\:Xg{h}_{h2}\:\:\:\:\:\:\:\:\:\:\:{x}_{9}=\varDelta\:{P}_{h}\:\:{x}_{10}=\int\:{ACE}_{h1}dt\:{x}_{11}=\int\:{ACE}_{h2}dt$$

Control vectors are:

$$\:{u}_{1}=\varDelta\:{P}_{Ch1}\:{u}_{2}=\varDelta\:{P}_{Ch2}$$

Disturbance vectors are:

$$\:{w}_{1}=\varDelta\:{P}_{dh1}\:\:\:\:\:\:\:\:\:\:\:\:\:\:\:\:\:{w}_{2}=\varDelta\:{P}_{dh2}$$

With reference to power system model as shown in Fig. 1, the following differential equations are derived.

12 $$\:\frac{d}{dt}\varDelta\:{F}_{h1}=\left(\varDelta\:P{g}_{h1}-\varDelta\:{P}_{h}-\varDelta\:P{d}_{h1}-\varDelta\:{P}_{dc1}\right)\left(K{p}_{h1}/T{p}_{h1}\right)-\left(1/\:{T}_{ph1}\right)\varDelta\:{F}_{h1}\:\:\:\:$$

Introducing parameters $\:{M}_{h1}$ and $\:{D}_{h1}$ and defining them as;

$$\:{M}_{h1}=T{p}_{h1}/K{p}_{h1}=\frac{2{H}_{h1}}{{f}^{0}}$$

$$\:{D}_{h1}=1/\:K{p}_{h1}$$

13 $$\:\frac{d}{dt}\varDelta\:{F}_{h1}=\left(\varDelta\:P{g}_{h1}-\varDelta\:{P}_{h}-\varDelta\:P{d}_{h1}-\varDelta\:{P}_{dc1}\right)\left(1/\:{M}_{h1}\right)-\left({D}_{h1}/{M}_{h1}\right)\varDelta\:{F}_{h1}$$

14 $$\:\frac{d}{dt}\varDelta\:P{g}_{h1}=\left(2/{\:\:T}_{w1}\right)\varDelta\:X{g}_{h1}-2\frac{d}{dt}\varDelta\:X{g}_{h1}-\left(2/{T}_{w1}\right)\varDelta\:P{g}_{h1}$$

15 $$\:\frac{d}{dt}\varDelta\:X{g}_{h1}=\left(1/{T}_{h3}\right)\varDelta\:Xg{h}_{h1}+\frac{d}{dt}\left({T}_{h1}/{T}_{h3}\right)\varDelta\:Xg{h}_{h1}-\left(1/{T}_{h3}\right)\varDelta\:X{g}_{h1}$$

16 $$\:\frac{d}{dt}\varDelta\:Xg{h}_{h1}=\varDelta\:P{c}_{h1}\left(K{g}_{h1}/T{g}_{h1}\right)-\varDelta\:{F}_{h1}\left(K{g}_{h1}/{R}_{h1}T{g}_{h1}\right)-\varDelta\:Xg{h}_{h1}/T{g}_{h1}$$

From Eqs. (14), (15) and (16), we can rewrite Eqs. (15) & (16) as:

17 $$\begin{aligned} \frac{d}{{dt}}\Delta Pg_{{h1}} & = \Delta F_{{h1}} \left( {2Kg_{{h1}} T_{{h1}} /R_{{h1}} T_{{h3}} Tg_{{h1}} } \right) - \Delta Pg_{{h1}} \left( {2/{\mathrm{~~}}T_{{w1}} } \right) + \Delta Xg_{{h1}} \left[ {\left( {2/T_{{w1}} } \right) + \left( {2/T_{{h3}} } \right)} \right] + \\ & \Delta Xgh_{{h1}} \left[ {\left( {2T_{{h1}} /T_{{h3}} Tg_{{h1}} } \right) - \left( {2/{\mathrm{~~}}T_{{h3}} } \right)} \right] - \Delta Pc_{{h1}} \left( {2Kg_{{h1}} T_{{h1}} /T_{{h3}} Tg_{{h1}} } \right) \\ \end{aligned}$$

18 $$\begin{aligned} \frac{d}{{dt}}\Delta Xg_{{h1}} & = - \Delta F_{{h1}} \left( {Kg_{{h1}} T_{{h1}} /R_{{h1}} T_{{h3}} Tg_{{h1}} } \right) - \Delta Xg_{{h1}} \left( {1/T_{{h3}} } \right) + \\ & \Delta Xgh_{{h1}} \left[ {\left( {1/{\mathrm{~~}}T_{{h3}} } \right) - \left( {T_{{h1}} /Tg_{{h1}} T_{{h3}} } \right)} \right] - \Delta Pc_{{h1}} \left( {Kg_{{h1}} T_{{h1}} /Tg_{{h1}} T_{{h3}} } \right) \\ \end{aligned}$$

Similarly for second area, the equations can be derived as;

19 $$\:\frac{d}{dt}\varDelta\:{F}_{h2}=\left(\varDelta\:P{g}_{h2}-{a}_{12}\varDelta\:{P}_{h}-\varDelta\:P{d}_{h2}-{a}_{12}\varDelta\:{P}_{dc}\right)\left(1/{\:M}_{h2}\right)-\left({D}_{h2}/{M}_{h2}\right)\varDelta\:{F}_{h2}$$

20 $$\begin{aligned} \frac{d}{{dt}}\Delta Pg_{{h2}} & = \Delta F_{{h2}} \left( {2Kg_{{h2}} T_{{h2}} /R_{{h2}} T_{{h4}} Tg_{{h2}} } \right) + \Delta Xg_{{h2}} \left[ {\left( {2/{\mathrm{~}}T_{{w2}} } \right) + \left( {2/T_{{h4}} } \right)} \right] - \left( {2/T_{{w2}} } \right)\Delta Pg_{{h2}} \\ & - \Delta Xgh_{{h2}} \left[ {\left( {2/T_{{h4}} } \right) - \left( {2T_{{h2}} /T_{{h4}} Tg_{{h2}} } \right)} \right] - \Delta Pc_{{h2}} \left( {2Kg_{{h2}} T_{{h2}} /T_{{h4}} Tg_{{h2}} } \right) \\ \end{aligned}$$

21 $$\begin{aligned} \frac{d}{{dt}}\Delta Xg_{{h2}} & = - \Delta F_{{h2}} \left( {Kg_{{h2}} T_{{h2}} /R_{{h2}} T_{{h4}} Tg_{{h2}} } \right) + \Delta Xgh_{{h2}} \left[ {\left( {1/{\mathrm{~}}T_{{h4}} } \right) - \left( {T_{{h2}} /Tg_{{h2}} T_{{h4}} } \right)} \right] \\ & - \left( {1/{\mathrm{~}}T_{{h4}} } \right)\Delta Xg_{{h2}} - \Delta Pc_{{h2}} \left( {Kg_{{h2}} T_{{h2}} /Tg_{{h2}} T_{{h4}} } \right) \\ \end{aligned}$$

22 $$\:\frac{d}{dt}\varDelta\:Xg{h}_{h2}=\varDelta\:P{c}_{h2}\left(K{g}_{h2}/T{g}_{h2}\right)-\varDelta\:{F}_{h2}\left(K{g}_{h2}/{R}_{h2}T{g}_{h2}\right)-\varDelta\:Xg{h}_{h2}/T{g}_{h2}$$

For the power system model as considered in this section, the equation can be rewritten as:

23 $$\:\frac{d}{dt}\varDelta\:{P}_{h}=2\pi\:{T}_{12}(\varDelta\:{F}_{h1}-\varDelta\:{F}_{h2})$$

and the expressions for the $\:\varDelta\:{P}_{h1}\:\&\:\varDelta\:{P}_{h2}$ can be obtained as:

24 $$\:\varDelta\:{P}_{h1}=\varDelta\:{P}_{h}$$

25 $$\:\varDelta\:{P}_{h2}=-\left({P}_{r1}/{P}_{r2}\right)\varDelta\:{P}_{h}={a}_{12}\varDelta\:{P}_{h}$$

Corresponding to the state variables $\:IAC{E}_{h1}$ & $\:IAC{E}_{h2}$, the $\:{10}^{th}$ and $\:{11}^{th}$ differential equations can be written as follows:

$$\:IAC{E}_{h1}=\int\:(\varDelta\:{P}_{h}+{B}_{h1}\varDelta\:{F}_{h1})dt$$

26 $$\:\frac{d}{dt}\left(IAC{E}_{h1}\right)=\varDelta\:{P}_{h}+{B}_{h1}\varDelta\:{F}_{h1}$$

$$\:IAC{E}_{h2}=\int\:(\varDelta\:{P}_{h2}+{B}_{h2}\varDelta\:{F}_{h2})dt$$

27 $$\:\frac{d}{dt}IAC{E}_{h2}=\left({a}_{12}\varDelta\:{P}_{h}\right)+{B}_{h2}\varDelta\:{F}_{h2}$$

The system matrices, $\:A,\:B$ and $\:C$ described by Eqs. (10) and (11) can be obtained with the structures of state, control and output vectors and the transfer function block diagram representation of Fig. 1. The corresponding original matrices and coefficient matrices are derived using the numerical values of system variables as given in the Appendix.

$$\:A=\left[\begin{array}{ccccccccccc}-\frac{{D}_{{h}_{1}}}{{M}_{{h}_{1}}}&\:\frac{1}{{M}_{{h}_{1}}}&\:0&\:0&\:0&\:0&\:0&\:0&\:-\frac{1}{{M}_{{h}_{1}}}&\:0&\:0\\\:\left(\frac{{2{K}_{g}}_{{h}_{1}}{T}_{{h}_{1}}}{{{R}_{{h}_{1}}{T}_{{h}_{1}}T}_{g{h}_{1}}}\right)&\:\left(-\frac{2}{{T}_{{w}_{1}}}\right)&\:\left(\frac{2}{{T}_{{w}_{1}}}+\frac{2}{{T}_{{h}_{3}}}\right)&\:\left(\frac{2{T}_{{h}_{1}}}{{{T}_{{h}_{3}}T}_{{gh}_{1}}}-\frac{2}{{T}_{{h}_{3}}}\right)&\:0&\:0&\:0&\:0&\:0&\:0&\:0\\\:\left(-\frac{{{K}_{g}}_{{h}_{1}}{T}_{{h}_{1}}}{{{R}_{{h}_{1}}{T}_{{h}_{1}}T}_{g{h}_{1}}}\right)&\:0&\:-\frac{1}{{T}_{{h}_{3}}}&\:\left(\frac{1}{{T}_{{h}_{3}}}-\frac{{T}_{{h}_{1}}}{{T}_{{gh}_{1}}{T}_{{h}_{3}}}\right)&\:0&\:0&\:0&\:0&\:0&\:0&\:0\\\:\left(-\frac{{{K}_{g}}_{{h}_{1}}}{{{R}_{{h}_{1}}T}_{g{h}_{1}}}\right)&\:0&\:0&\:-\frac{1}{{T}_{{gh}_{1}}}&\:0&\:0&\:0&\:0&\:0&\:0&\:0\\\:0&\:0&\:0&\:0&\:-\frac{{D}_{{h}_{2}}}{{M}_{{h}_{2}}}&\:\frac{1}{{M}_{{h}_{2}}}&\:0&\:0&\:0&\:0&\:0\\\:0&\:0&\:0&\:0&\:\left(\frac{{2{K}_{g}}_{{h}_{2}}{T}_{{h}_{2}}}{{{R}_{{h}_{2}}{T}_{{h}_{4}}T}_{g{h}_{2}}}\right)&\:\left(-\frac{2}{{T}_{{w}_{2}}}\right)&\:\left(\frac{2}{{T}_{{w}_{2}}}+\frac{2}{{T}_{{h}_{4}}}\right)&\:\left(\frac{-2}{{T}_{{h}_{4}}}-\frac{{2T}_{{h}_{2}}}{{T}_{{gh}_{2}}{T}_{{h}_{4}}}\right)&\:0&\:0&\:0\\\:0&\:0&\:0&\:0&\:\left(-\frac{{{K}_{g}}_{{h}_{2}}{T}_{{h}_{2}}}{{{R}_{{h}_{2}}{T}_{{h}_{4}}T}_{g{h}_{2}}}\right)&\:0&\:-\frac{1}{{T}_{{h}_{4}}}&\:\left(\frac{1}{{T}_{{h}_{4}}}-\frac{{T}_{{h}_{2}}}{{T}_{{gh}_{2}}{T}_{{h}_{4}}}\right)&\:0&\:0&\:0\\\:0&\:0&\:0&\:0&\:\left(-\frac{{{K}_{g}}_{{h}_{2}}}{{{R}_{{h}_{2}}T}_{g{h}_{2}}}\right)&\:0&\:0&\:-\frac{1}{{T}_{{gh}_{2}}}&\:0&\:0&\:0\\\:2\pi\:{T}_{12}&\:0&\:0&\:0&\:-2\pi\:{T}_{12}&\:0&\:0&\:0&\:0&\:0&\:0\\\:{B}_{{h}_{1}}&\:0&\:0&\:0&\:0&\:0&\:0&\:0&\:1&\:0&\:0\\\:0&\:0&\:0&\:0&\:{B}_{{h}_{2}}&\:0&\:0&\:0&\:{a}_{12}&\:0&\:0\end{array}\right]$$

$$\:B=\left[\begin{array}{cc}0&\:0\\\:\left(-\frac{{2{K}_{g}}_{{h}_{1}}{T}_{{h}_{1}}}{{{T}_{{h}_{3}}T}_{g{h}_{1}}}\right)&\:0\\\:\left(\frac{{{K}_{g}}_{{h}_{1}}{T}_{{h}_{1}}}{{{T}_{{h}_{3}}T}_{g{h}_{1}}}\right)&\:0\\\:\left(\frac{{{K}_{g}}_{{h}_{1}}}{{T}_{g{h}_{1}}}\right)&\:0\\\:0&\:0\\\:0&\:\left(-\frac{{2{K}_{g}}_{{h}_{2}}{T}_{{h}_{2}}}{{{T}_{{h}_{4}}T}_{g{h}_{2}}}\right)\\\:0&\:\left(-\frac{{{K}_{g}}_{{h}_{2}}{T}_{{h}_{2}}}{{{T}_{{h}_{4}}T}_{g{h}_{2}}}\right)\\\:0&\:\left(\frac{{{K}_{g}}_{{h}_{2}}}{{T}_{g{h}_{2}}}\right)\\\:0&\:0\\\:0&\:0\\\:0&\:0\end{array}\right]$$

The coefficient matrices based on these system data may be obtained as follows;

$$\:A=\left[\begin{array}{ccccccccccc}-0.05\:&\:5.988\:&\:0&\:0&\:0&\:0&\:0&\:0&\:-5.988&\:0&\:0\\\:1.623&\:-2.0&\:2.41&\:0.362&\:0&\:0&\:0&\:0&\:0&\:0&\:0\\\:-0.8&\:0&\:-0.0205&\:-0.1796&\:0&\:0&\:0&\:0&\:0&\:0&\:0\\\:-0.812&\:0&\:0&\:-1.949&\:0&\:0&\:0&\:0&\:0&\:0&\:0\\\:0&\:0&\:0&\:0&\:-0.05&\:5.988&\:0&\:0&\:5.988&\:0&\:0\\\:0&\:0&\:0&\:0&\:0.1667&\:-2&\:2.041&\:0.362&\:0&\:0&\:0\\\:0&\:0&\:0&\:0&\:-0.0834&\:0&\:-0.0205&\:-0.1796&\:0&\:0&\:0\\\:0&\:0&\:0&\:0&\:-0.812&\:0&\:0&\:-1.949&\:0&\:0&\:0\\\:0.545&\:0&\:0&\:0&\:-0.545&\:0&\:0&\:0&\:0&\:0&\:0\\\:0.425&\:0&\:0&\:0&\:0&\:0&\:0&\:0&\:1&\:0&\:0\\\:0&\:0&\:0&\:0&\:0.425&\:0&\:0&\:0&\:-1&\:0&\:0\end{array}\right]$$

$$\:B=\left[\begin{array}{cc}0&\:0.0\\\:-3.896&\:0.0\\\:1.94&\:0.0\\\:0.20&\:0.0\\\:0.0&\:0.0\\\:0.0&\:-3.896\\\:0.0&\:-1.94\\\:0.0&\:0.2\\\:0.0&\:0.0\\\:0.0&\:0.0\\\:0.0&\:0.0\end{array}\right];$$

$$\:C=\left[\begin{array}{ccccccccccc}1&\:0&\:0&\:0&\:0&\:0&\:0&\:0&\:0&\:0&\:0\\\:0&\:0&\:0&\:0&\:1&\:0&\:0&\:0&\:0&\:0&\:0\\\:0&\:0&\:0&\:0&\:0&\:0&\:0&\:0&\:1&\:0&\:0\end{array}\right];\:D=\left[0\right]$$

Eigen values of open loop transfer function i.e. for the system matrix A are given in Table 1. As evident from the table, the system is unstable in open loop configuration. An optimal AGC regulator is designed using full state vector feedback control strategy.

The optimal gains (*K*) for the system are obtained as;

$$\:K=\left[\begin{array}{ccccccccccc}0.11&\:-0.14&\:-3&\:0.2&\:0.59&\:1.61&\:1.4&\:0.14&\:1.68&\:-0.44&\:-0.89\\\:0.49&\:2.45&\:5.02&\:0.12&\:1.48&\:1.69&\:1.14&\:0.02&\:-2.75&\:\mathrm{0,89}&\:0.44\end{array}\right]$$

Closed loop matrix $\:{A}_{c}$ is given by;

$$\:{A}_{c}=A-BK$$

$$\:{A}_{c}=\left[\begin{array}{ccccccccccc}0.15&\:6.97&\:2.01&\:0.05&\:0.59&\:0.68&\:0.46&\:0.01&\:-7.09&\:0.36&\:0.18\\\:0.17&\:-2.0&\:2.04&\:0.36&\:0&\:0&\:0&\:0&\:0&\:0&\:0\\\:-0.14&\:-0.55&\:-2.23&\:-0.12&\:-0.06&\:0.31&\:0.33&\:0.05&\:1.22&\:-0.36&\:0.27\\\:-0.81&\:0&\:0&\:-1.95&\:0&\:0&\:0&\:0&\:0&\:0&\:0\\\:-0.98&\:-4.75&\:-9.17&\:-0.28&\:-3.06&\:2.37&\:-2.51&\:-0.07&\:11.02&\:-1.66&\:1.05\\\:0&\:0&\:0&\:0&\:0.17&\:-2.00&\:2.04&\:0.36&\:0&\:0&\:0\\\:-0.21&\:0.28&\:5.85&\:-0.39&\:-1.24&\:-3.14&\:-2.75&\:-0.46&\:-3.28&\:0.87&\:-1.74\\\:0&\:0&\:0&\:0&\:-0.81&\:0&\:0&\:-1.95&\:0&\:0&\:0\\\:0.55&\:0&\:0&\:0&\:-0.55&\:0&\:0&\:0&\:0&\:0&\:0\\\:0.43&\:0&\:0&\:0&\:0&\:0&\:0&\:0&\:1&\:0&\:0\\\:0&\:0&\:0&\:0&\:0.43&\:0&\:0&\:0&\:-1&\:0&\:0\end{array}\right]$$

$$\:B=\left[\begin{array}{cc}0&\:0.0\\\:-3.896&\:0.0\\\:1.94&\:0.0\\\:0.20&\:0.0\\\:0.0&\:0.0\\\:0.0&\:-3.896\\\:0.0&\:1.94\\\:0.0&\:0.2\\\:0.0&\:0.0\\\:0.0&\:0.0\\\:0.0&\:0.0\end{array}\right]\:;\:C=\left[\begin{array}{ccccccccccc}1&\:0&\:0&\:0&\:0&\:0&\:0&\:0&\:0&\:0&\:0\\\:0&\:0&\:0&\:0&\:1&\:0&\:0&\:0&\:0&\:0&\:0\\\:0&\:0&\:0&\:0&\:0&\:0&\:0&\:0&\:1&\:0&\:0\end{array}\right];\:D=\left[0\right]$$

The Hankel singular values of the system are: $\left[0.7239\:\:\:0.5565\:\:\:0.4006\:\:\:0.3851\:\:\:0.2131\:\:\:0.1382\:\:\:0.1041\:\:\:0.0276\:\:\:0.0156\:\:0.0017\:\:\:0.0001\right]$

**Fig. 2:**
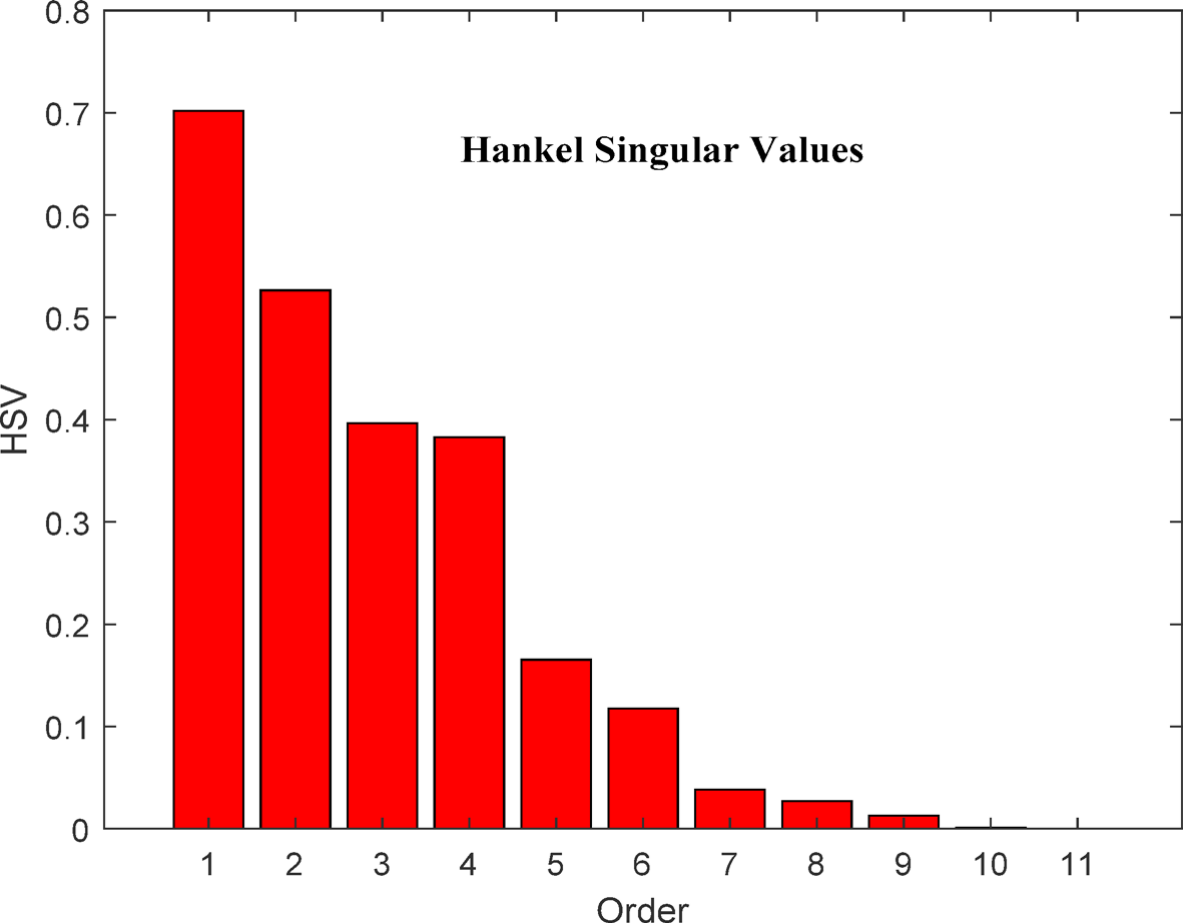
Bar chart of 11th order model.

**Fig. 3:**
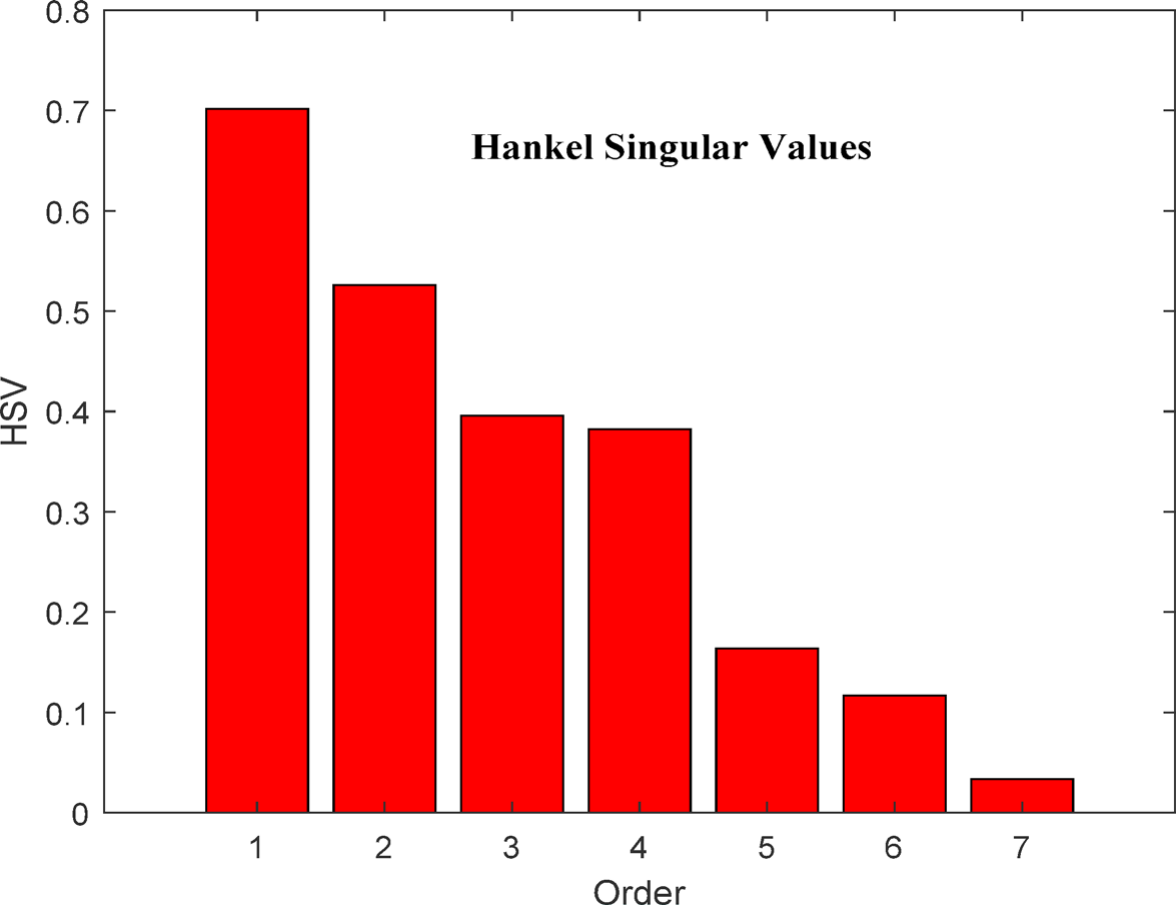
Bar chart of 7th order model.

A bar chart of the Hankel singular values for the original system is shown in Fig. 2, illustrating the energy levels of the system states. Lower eigenvalues correspond to high-energy states. In the reduced system, high-energy states are preserved, while low-energy states can be omitted. As seen in Fig. 3, the system can effectively be reduced to a seventh-order model.

Consequently, a seventh, eighth and ninth order approximations are derived using MATLAB software.

$$\:{A}_{r7}=\left[\begin{array}{ccccccc}-1.054&\:1.875&\:0.7166&\:-0.6242&\:0.6939&\:-1.049&\:0.1107\\\:-3.388&\:-1.054&\:0.4565&\:-1.27&\:0.5124&\:-1.013&\:0.5481\\\:0&\:0&\:-0.8093&\:-1.484&\:-0.4097&\:0.4946&\:0.5085\\\:0&\:0&\:1.136&\:-0.8093&\:-0.01636&\:-0.3323&\:0.8419\\\:0&\:0&\:0&\:0&\:-0.3794&\:0.7792&\:0.02083\\\:0&\:0&\:0&\:0&\:-0.153&\:-0.3794&\:0.1043\\\:0&\:0&\:0&\:0&\:0&\:0&\:-0.2723\end{array}\right]$$

$$\:{B}_{r7}=\left[\begin{array}{cc}-0.2439&\:0.3926\\\:0.04211&\:0.3109\\\:0.2248&\:-0.05598\\\:0.1774&\:\:0.5127\\\:0.2176&\:-0.1061\\\:-0.1608&\:0.1566\\\:-0.1042&\:-0.197\end{array}\right]$$

$$\:{C}_{r7}=\left[\begin{array}{ccccccc}-1.231&\:\:-0.4024&\:-1.337&\:0.061&\:0.04303&\:-0.08817&\:0.01245\\\:-0.09541&\:0.04648&\:0.06692&\:0.02051&\:-0.3688\:\:\:\:&\:0.2971&\:-0.01824\\\:1.385&\:1.872&\:-0.6518&\:0.8895&\:-0.03136&\:0.6425&\:-0.3018\\\:-0.4591&\:0.1342&\:0.6414&\:0.771&\:0.5888&\:-0.3517\:\:\:&\:-0.3588\\\:-0.3696&\:0.5467&\:0.1221&\:-0.2155&\:-0.3576\:\:\:\:&\:0.2984&\:-0.0608\end{array}\right]$$

$$\:{D}_{r7}=\left[0\right]$$

## Balanced truncation method

This method involves balancing the system’s controllability and observability to identify dominant modes. By keeping only the most significant modes, it constructs a reduced-order model while minimizing error. The method of balanced truncation can be applied to linear MIMO systems.

Following are the main steps to find reduced order model using Balanced truncation.

Step 1- Find controllability grammian and obsevability grammian ($\:{W}_{o})$.

Step 2- Calculate a nonsingular matrix $\:{L}_{c}\in\:{\mathbb{R}}^{\mathrm{r}\times\:\mathrm{m}}$ such that

$$\:{W}_{c}={{{L}_{c}}^{T}L}_{c}.$$

Step 3- Find singular value decomposition of the product as;

$$\:{L}_{c}{W}_{c}{{L}_{c}}^{T}={USU}^{T}$$

$$\:\mathrm{W}\mathrm{h}\mathrm{e}\mathrm{r}\mathrm{e}\:{UU}^{T}=1\:\mathrm{a}\mathrm{n}\mathrm{d}\:S=diag({\sigma\:}_{1,\:\:}{\sigma\:}_{2},\dots\:\dots\:\dots\:{\sigma\:}_{n})>0$$

Where $\:{\sigma\:}_{1,\:\:}{\sigma\:}_{2},\dots\:$ are the singular values.

Step 4-Find the non-singular matrix for linear transformation.

Step 5- Find the linear transformation of the state space matrices A, B,C & D.

Step 6- Obtain the reduced model;

which shows that the transformed system is in balanced coordinates.

The reduced seventh order model obtained using truncation as;

$$\:{A}_{r7}=\left[\begin{array}{ccccccc}-0.1773&\:2.1266&\:-0.5438&\:0.1886&\:0.3649&\:0.1324&\:0.1415\\\:-2.2537&\:-0.7083&\:-0.3348&\:-0.5137&\:-0.4167&\:-0.1975&\:0.1196\\\:0.9707&\:1.8348&\:-1.4450&\:1.0471&\:1.3156&\:-0.6777&\:-0.8088\\\:-0.1050&\:0.3834&\:-1.3058&\:-0.0855&\:0.1846&\:-0.3229&\:-0.1781\\\:-0.5235&\:-0.7442&\:0.3872&\:-0.2934&\:-1.0031&\:-0.3509&\:0.1609\\\:0.1223&\:0.3413&\:0.0802&\:0.3097&\:1.1807&\:-0.4563&\:-0.3869\\\:-0.0347&\:-0.1032&\:0.6552&\:0.2628&\:0.2763&\:-0.3446&\:-0.5025\end{array}\right]$$

$$\:{B}_{r7}=\left[\begin{array}{cc}0.0963&\:-0.4973\\\:0.0863&\:-0.8837\\\:0.3006&\:1.0331\\\:0.2531&\:0.0423\\\:0.3800&\:-0.5320\\\:-0.3205&\:0.1528\\\:-0.3130&\:-0.0815\end{array}\right]$$

$$\:{C}_{r7}=\left[\begin{array}{ccccccc}-0.4768&\:0.6861&\:-0.0071&\:0.2306&\:0.2363&\:0.2540&\:0.1516\\\:-0.0138&\:-0.5254&\:1.0717&\:0.0717&\:-0.5425&\:0.2386&\:0.2516\\\:0.1706&\:0.2039&\:0.0949&\:0.0869&\:-0.2781&\:0.0681&\:-0.1354\end{array}\right]$$

& $$\:D=\left[0\right]$$.

## Analysis of results

**Fig. 4:**
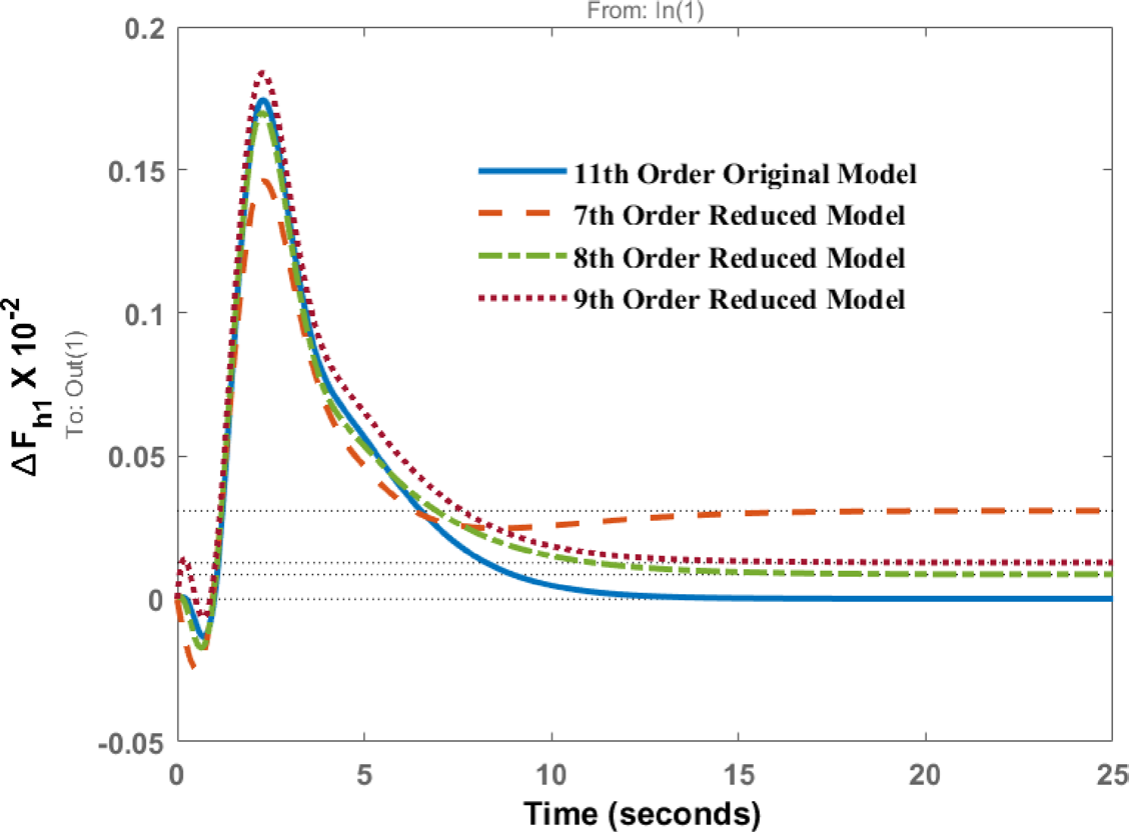
Dynamic behavior of change in frequency at area 1 for disturbance in area 1.

**Fig. 5:**
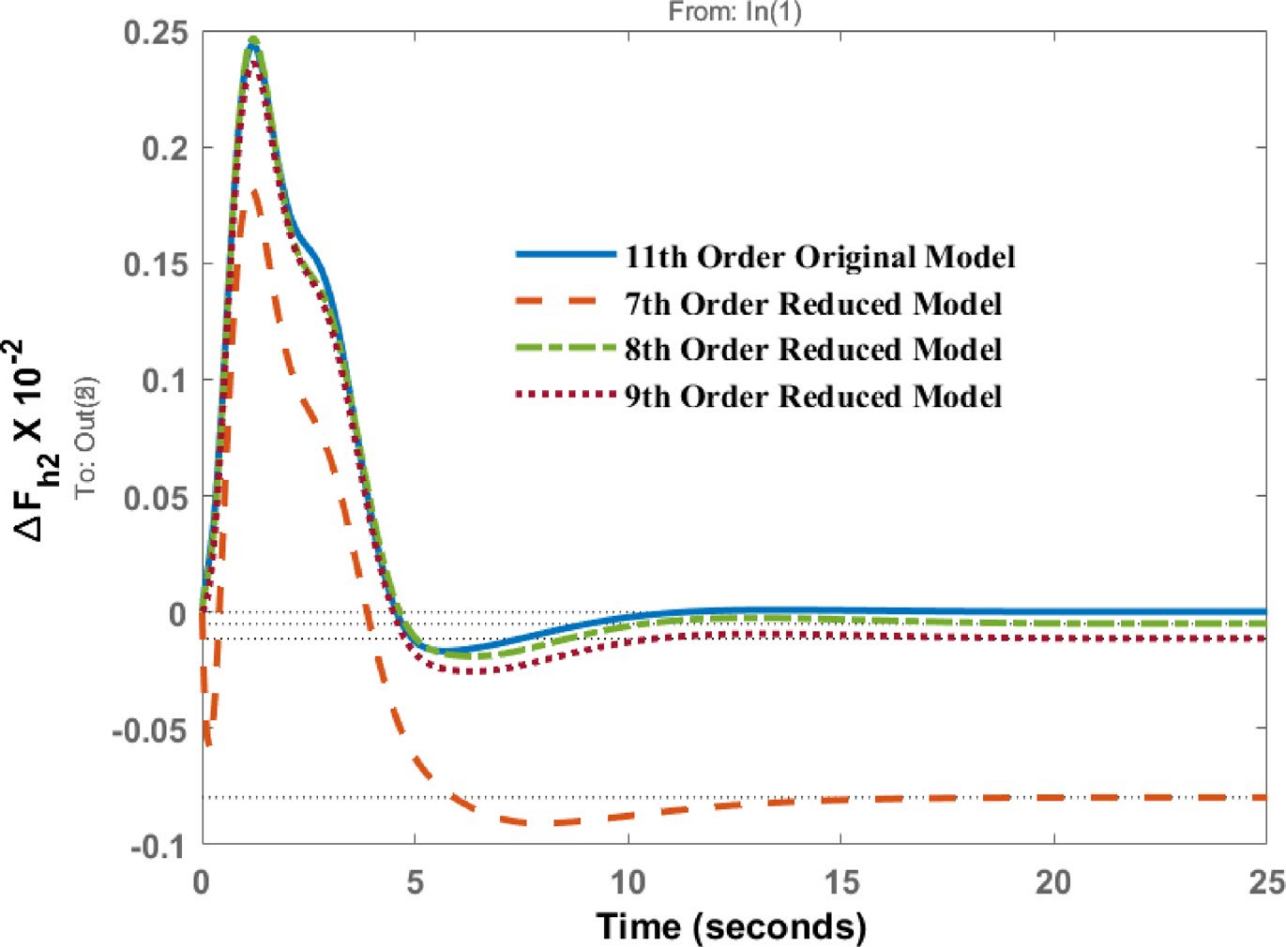
Dynamic behavior of change in frequency at area 2 for disturbance in area 1.

**Fig. 6:**
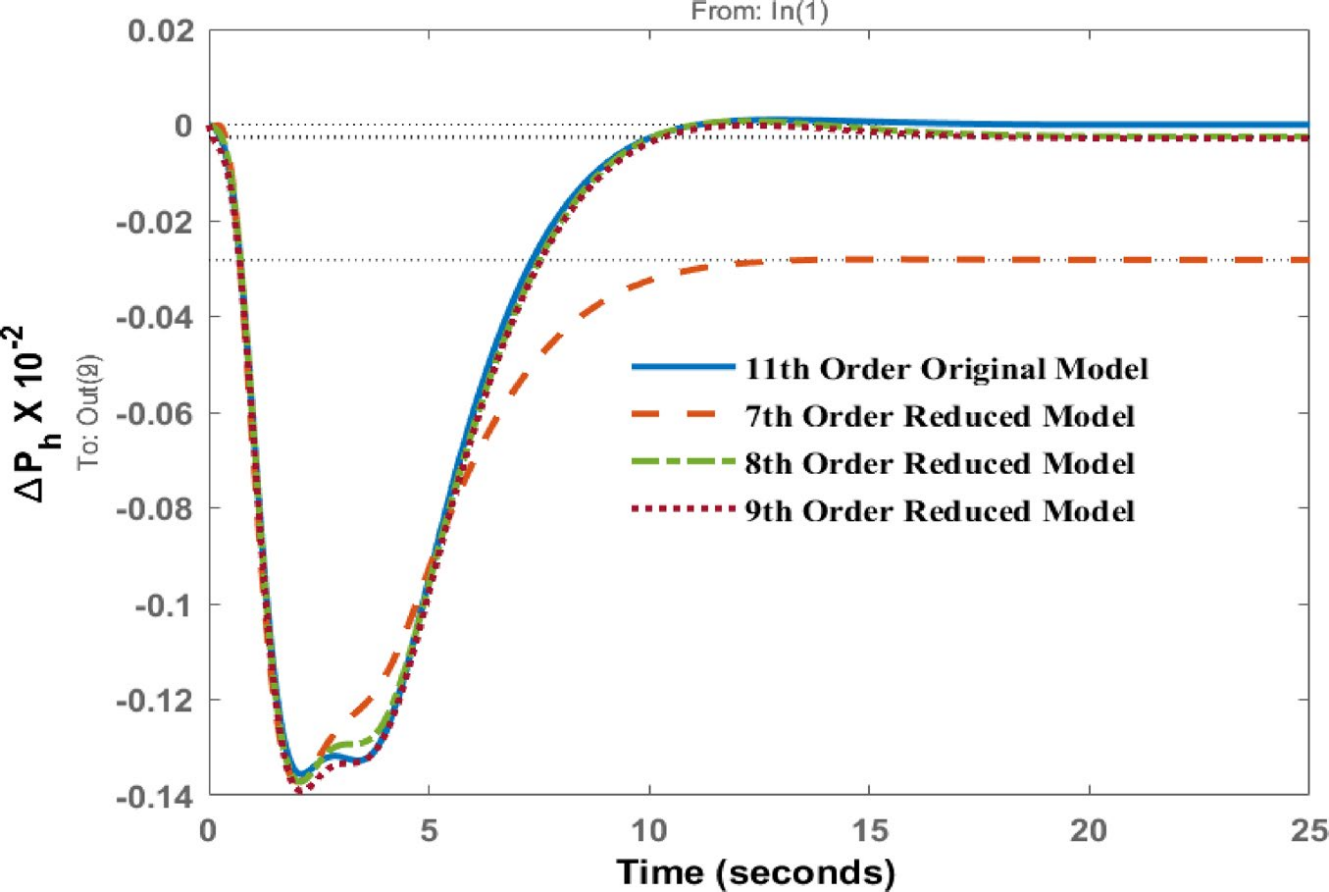
Dynamic behavior of $\:\varDelta\:{P}_{h}$ for disturbance in area 1.

**Fig. 7:**
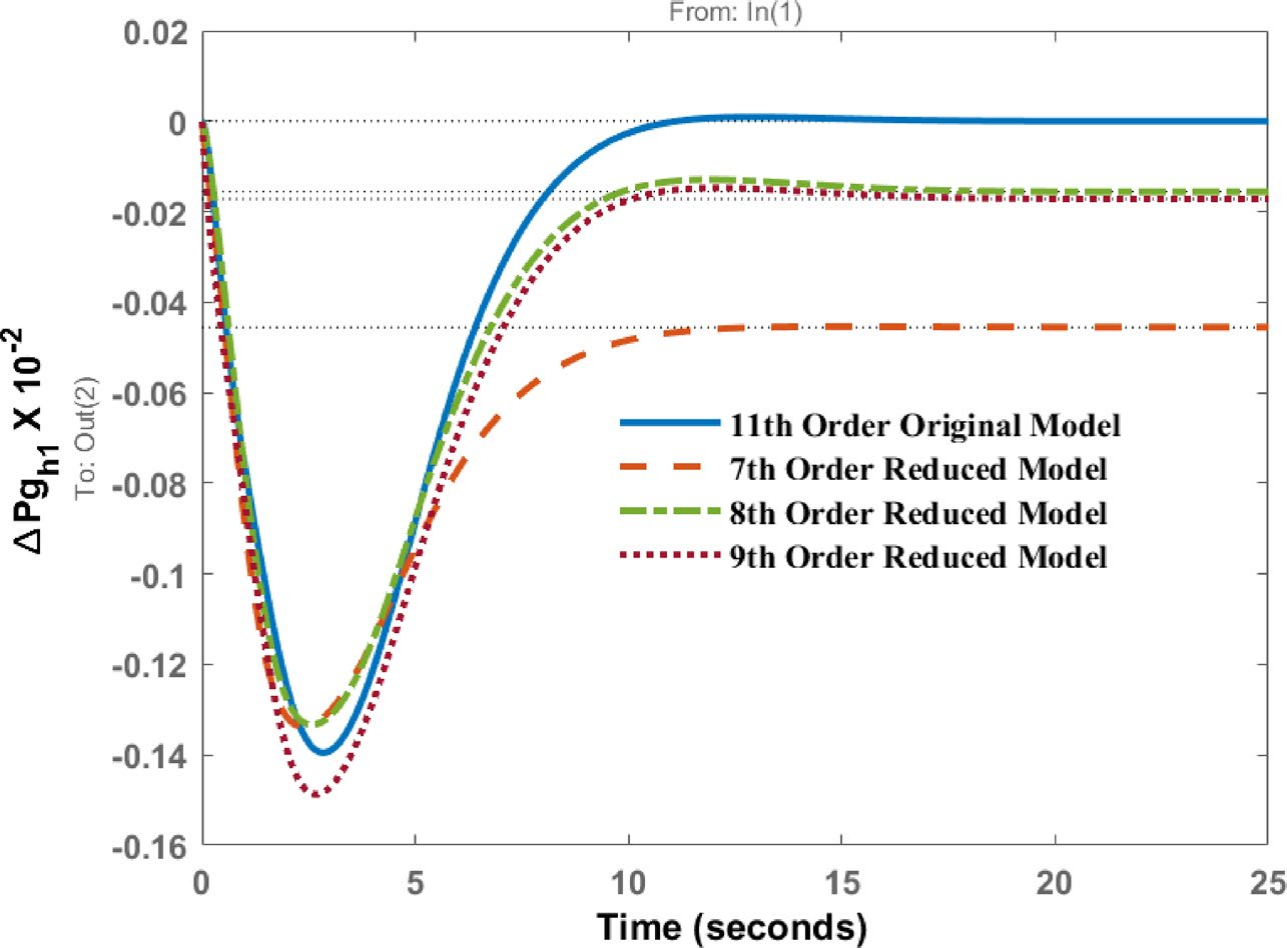
Dynamic behavior of change in Governor power at area 1 for disturbance in area 1.

**Fig. 8:**
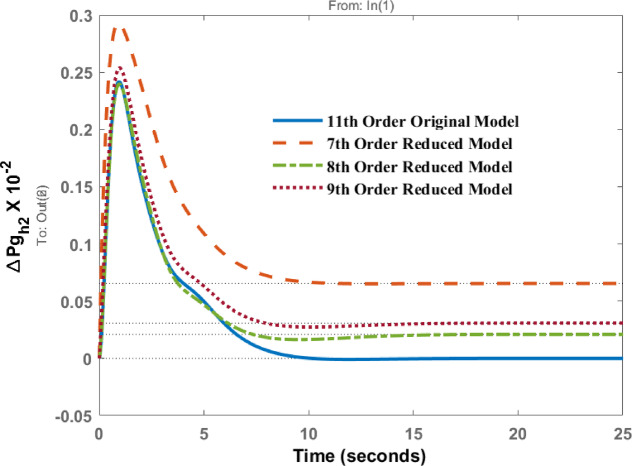
Dynamic behavior of change in Governor power at area 2 for disturbance in area 1.

**Fig. 9:**
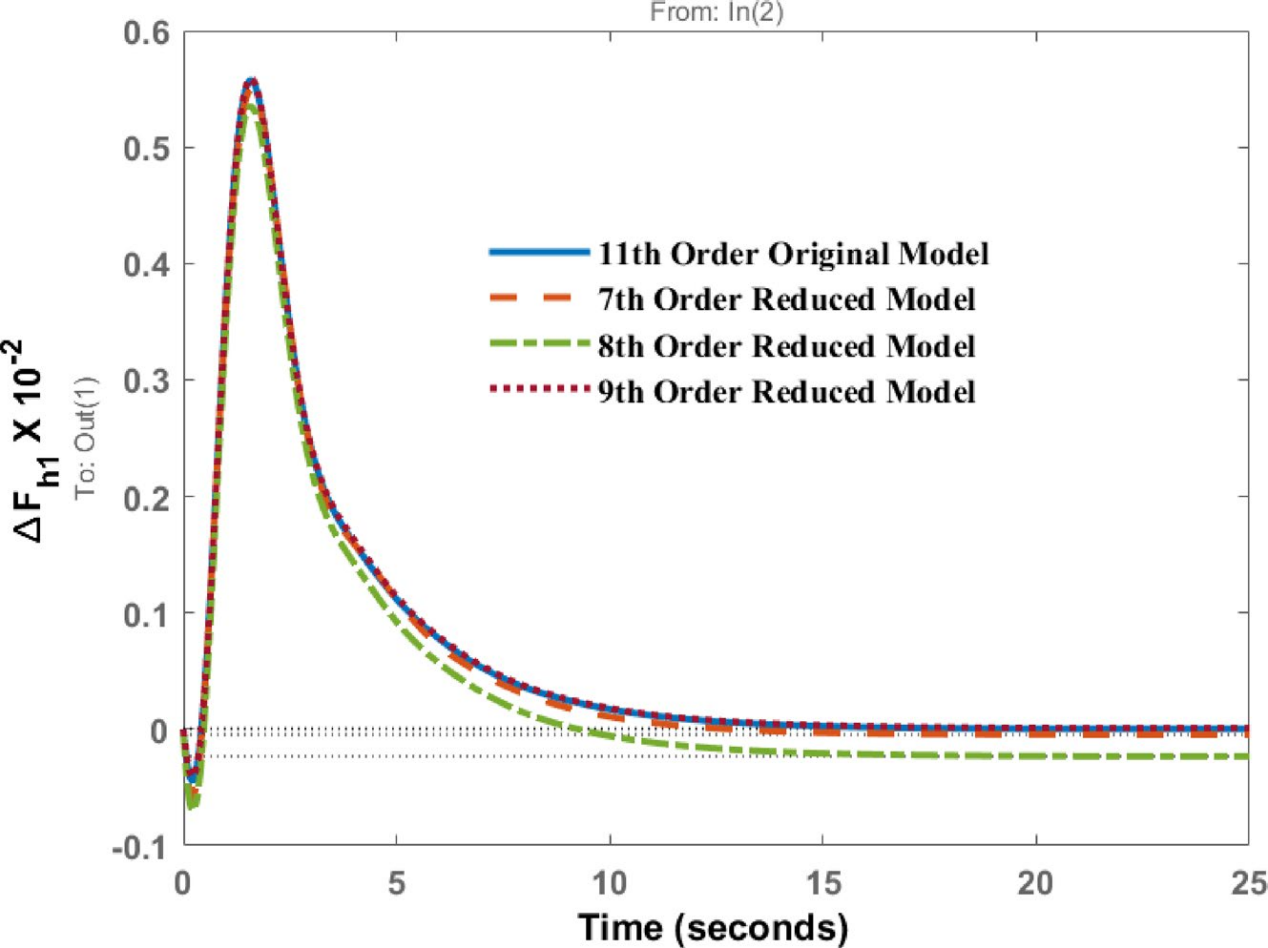
Dynamic behavior of change in frequency at area 1 for disturbance in area 2.

**Fig. 10:**
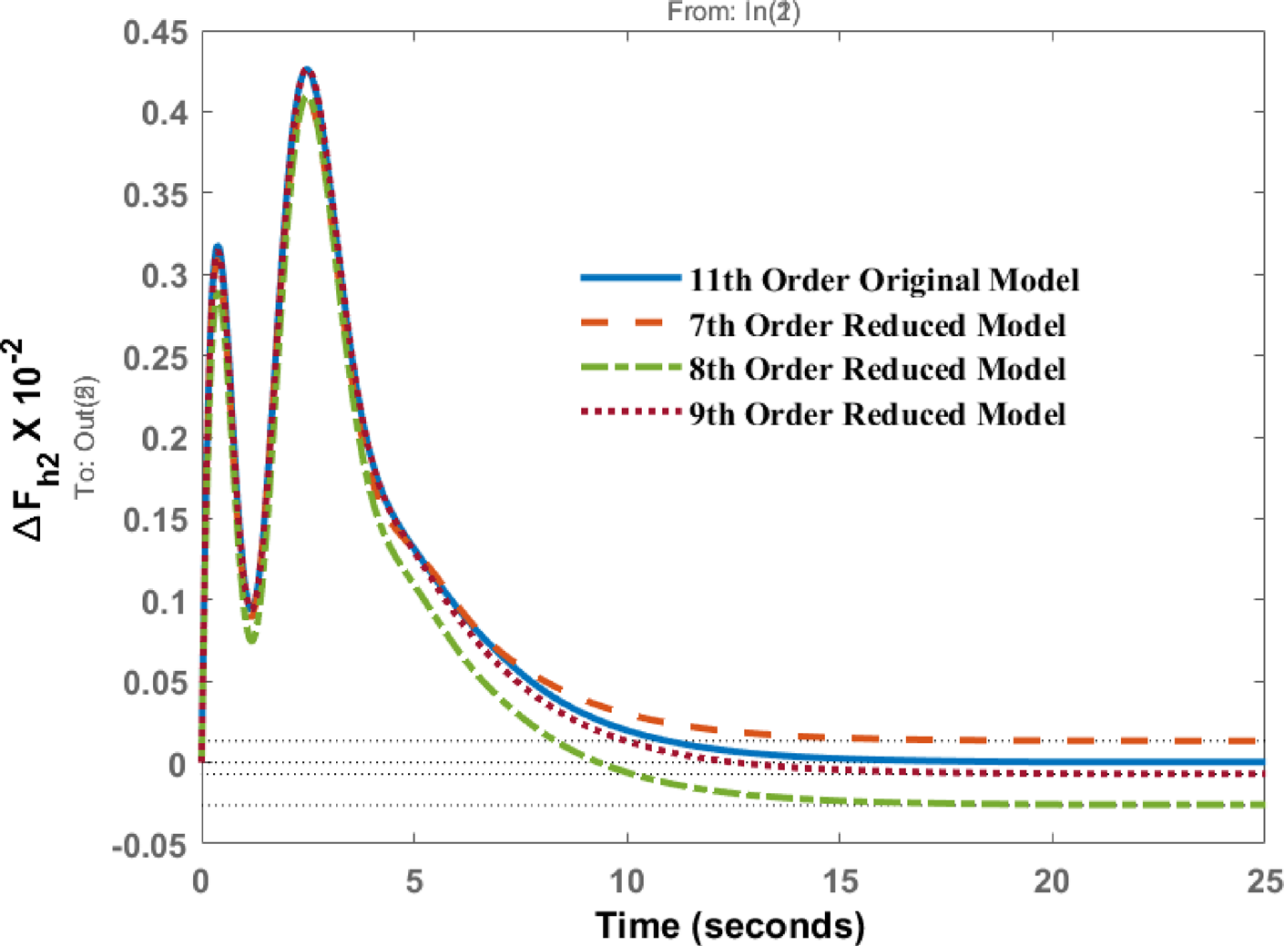
Dynamic behavior of change in frequency at area 2 for disturbance in area 2.

**Fig. 11:**
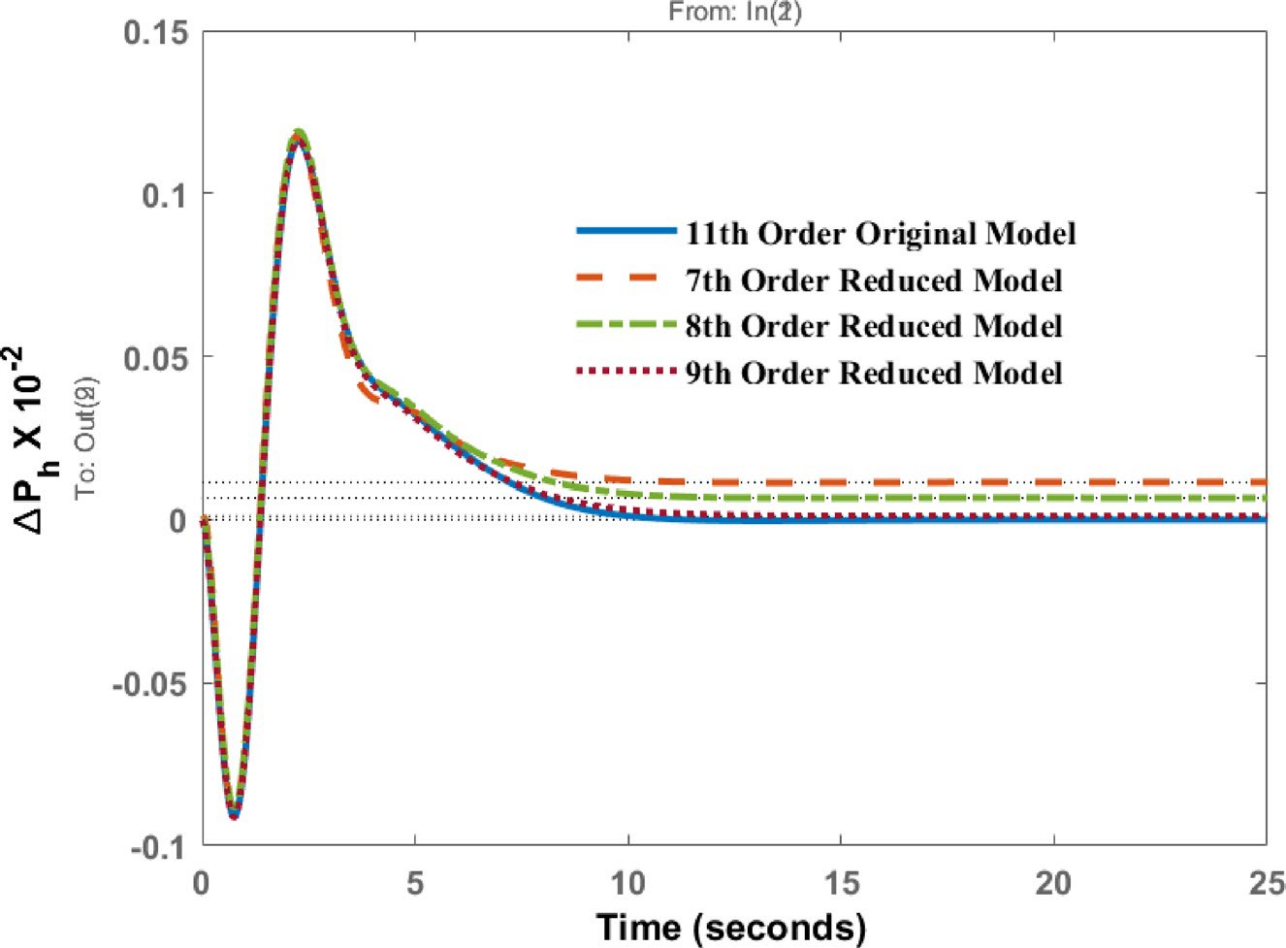
Dynamic behavior of $\:\varDelta\:{P}_{h}$ for disturbance in area 2.

**Fig. 12:**
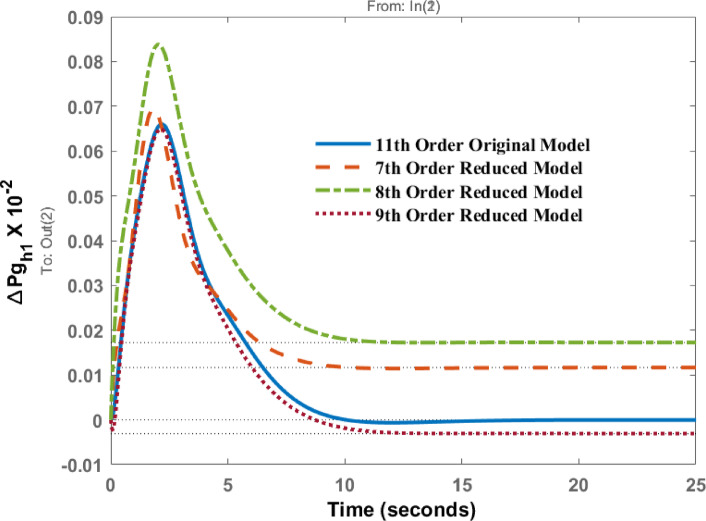
Dynamic behavior of change in Governor power at area 1 for disturbance in area 2.

**Fig. 13:**
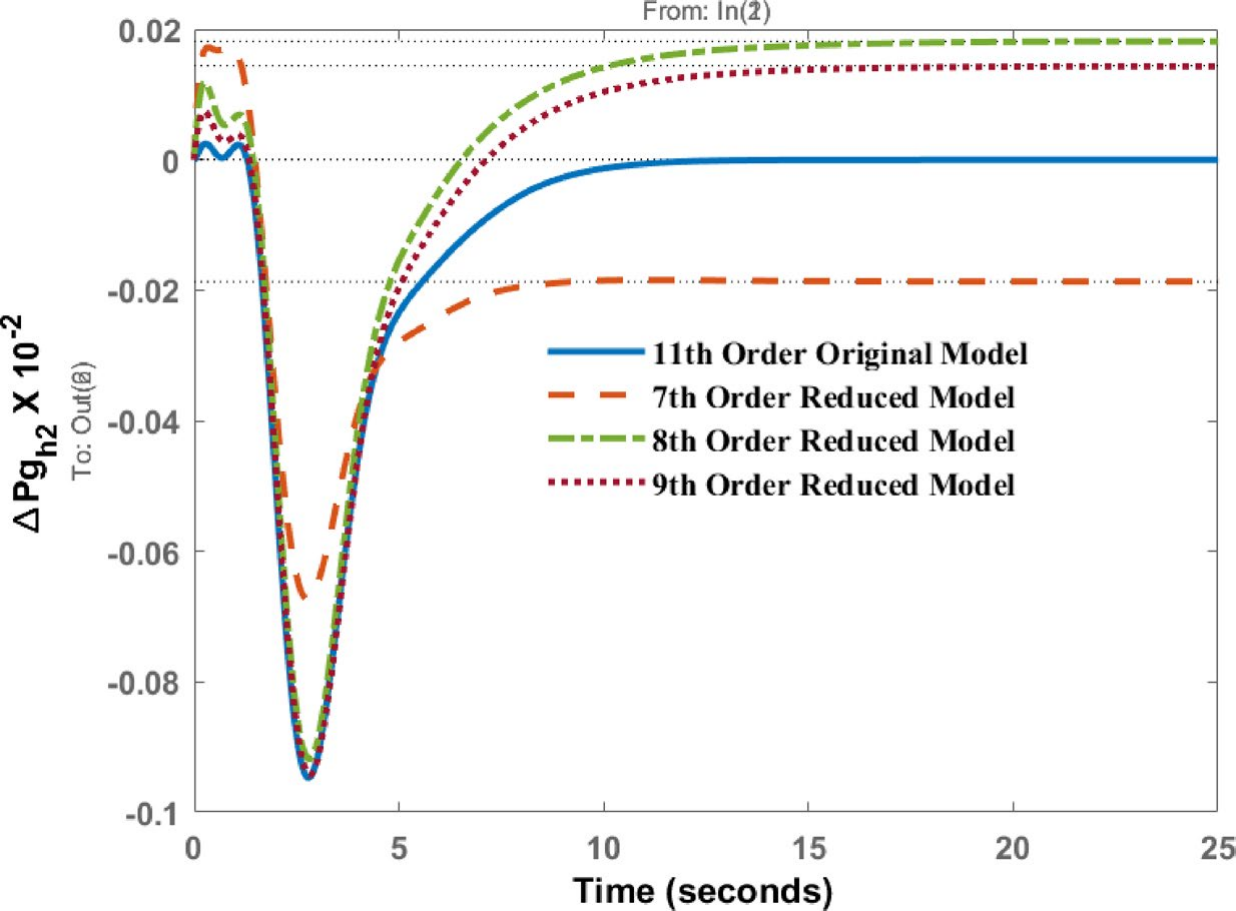
Dynamic behavior of change in Governor power at area 2 for disturbance in area 2.

The AGC problem of hydro governing system is evaluated and presented using HNA based approach. The simulation results of the original model i.e., 11th order model and reduced order models such as 09th order, 08th order and 07th order are presented through Figs. 4–13 for a step load disturbance. The energy states of the eigenvalues of the original higher-order model are shown in Fig. 2, while those of the reduced model are depicted in Fig. 3. To examine the dynamic behaviour of the system and validate the reduced order model obtained using the HNA method, the responses of these models are compared for different states of the system.

The bar chart in Fig. 2 illustrates the energy levels of different states of the original 11th -order system. It is evident from the chart that states 1–7 are high-energy states, indicating that the system can be successfully reduced to as low as the 7th order. Therefore, the system under consideration is reduced to the 7th, 8th, and 9th order models. In addition, the bar chart in Fig. 3 shows the energy states retained in the reduced 7th -order model. The high-energy states are preserved in the reduced model, while the last four states of the original model are eliminated in this study. The high-energy states correspond to the lower eigenvalues. The characteristic of original and reduced order model based on the dynamic response is tabulated in Table 1. In Table 1 ‘A’ represents the amplitude and ‘t’ the time of peak response characteristics, ‘t_s_’ represents settling time and ‘Ss’ represents steady state value of the response. Figure 4 represent the deviation in frequency of Area 1 when unit step disturbance is applied at Area 1. It is evident from the response that 8th order, and 9th order reduced model almost coincides with the response of original 11th order model. However, for 7th order model the amplitude is little bit smaller than original one. Steady state response is however comparable. This shows that as we reduce the order to lowest, response is deviated from original one.

The frequency deviation in area 2 for a disturbance in area 1 is shown in Fig. 5. A comparison of the dynamic responses of the 7th, 8th, and 9th order models indicates that the amplitudes of the original and reduced models occur at approximately the same time, 1.19 s, with comparable magnitudes. From Fig. 5, it is evident that the response of 8th order and 9th order model shows the same dynamic behaviour, but 7th order response follows the same response initially, but after peak overshoot there is a deviation from the original response and there exist steady state error in the system response and this cannot be appreciated. The steady state response of 8th and 9th order is comparable with original one.

**Table 1:** Comparison of characteristics of original 11th order with 9, 8 and 7th order.

Characteristic parameter	$\:\varDelta\:{\boldsymbol{F}}_{\boldsymbol{h}1}$ for disturbance at area 1(Fig. 4)				$\:\varDelta\:{\boldsymbol{F}}_{\boldsymbol{h}1}$ for disturbance at area 2 (Fig. 9)			
	Original model (11 order)	Reduced model (9 order)	Reduced model (8 order)	Reduced model (7 order)	Original model (11 order)	Reduced model (9 order)	Reduced model (8 order)	Reduced model (7 order)
$\:\boldsymbol{A}$ $\:\boldsymbol{t}\:$(s)	0.17 2.3	0.174 2.28	0.183 2.29	0.17 2.33	0.557 1.58	0.55 1.59	0.544 1.61	0.553 1.61
$\:{\boldsymbol{t}}_{\boldsymbol{s}}$ (s)	10.5	10.5	12.9	15.6	11.2	11	12.1	13.4
$\:{\boldsymbol{S}}_{\boldsymbol{s}}$	0.0	0.0009	0.006	-0.0119	0.0	-0.00104	-0.0163	-0.0122

	$\:\varDelta\:{\boldsymbol{F}}_{\boldsymbol{h}2}$ for disturbance at area 1 (Fig. 5)				$\:\varDelta\:{\boldsymbol{F}}_{\boldsymbol{h}2}$ for disturbance at area 2 (Fig. 10)			
$\:\boldsymbol{A}$ $\:\boldsymbol{t}\:$(s)	0.244 1.19	0.244 1.2	0.239 1.19	0.213 1.16	0.426 2.48	0.427 2.48	0.431 2.46	0.418 2.51
$\:{\boldsymbol{t}}_{\boldsymbol{s}}$ (s)	9.1	9.2	14.2	15.1	12	11.9	12.4	13.5
$\:{\boldsymbol{S}}_{\boldsymbol{s}}$	0.0	0.00029	-0.0131	-0.0484	0	-0.00018	-0.0006	-0.0159

	$\:\varDelta\:\boldsymbol{P}\boldsymbol{h}$ for disturbance at area 1 (Fig. 6)				$\:\varDelta\:\boldsymbol{P}\boldsymbol{h}$ for disturbance at area 2 (Fig. 11)			
$\:\boldsymbol{A}$ $\:\boldsymbol{t}\:$(s)	-0.136 2.11	-0.136 2.1	-0.137 2.12	-0.136 2.15	0.116 2.27	0.116 2.28	0.117 2.29	0.117 2.24
$\:{\boldsymbol{t}}_{\boldsymbol{s}}$ (s)	10	9.89	13.9	12	9.34	9.19	12.1	14
$\:{\boldsymbol{S}}_{\boldsymbol{s}}$	0.0	0.00008	-0.012	-0.0079	0.0	0.0013	0.00961	0.0225

The change in tie line power $\:\varDelta\:{P}_{h}$ is presented in Fig. 6 for the disturbance in area 1. The transient behavior of all models is similar. Around the initial dip (first 5 s), all models show a negative transient response before recovering to the steady state. The eighth- and ninth-order models track the original model very closely, nearly overlapping. However, the steady-state response of the seventh-order model is not comparable and shows the considerable amount of steady state error in the AGC response.

Deviations in governor power of area 1 known as $\:\varDelta\:P{g}_{h1}$and area 2 as $\:\varDelta\:P{g}_{h2}$ due to disturbance in area 1 are represented in Figs. 7 and 8 respectively. The responses show the identical dynamic behaviour and comparable static behaviour.

Frequency deviations of area 1( $\:{\varDelta\:F}_{h1})$ and area 2 $\:{(\varDelta\:F}_{h2})\:$for a disturbance in area 2 are shown in Figs. 9 and 10. It is observed that the responses of the 7th, 8th, and 9th order models closely follow that of the original model. For ΔF_h2_, the settling time of the original model is 12 s, for the 8th order model it is 12.4 s, and for the 7th order model it is 13.5 s, which is slightly higher.

Deviation in tie line power $\:{\varDelta\:P}_{h}\:$ is observed in Fig. 11 for disturbance in area 2. Peak values are similar for original and 7th, 8th and 9th order model around 2.27 s. These results shows that high energy states are retained in 7th, 8th and 9th order model, that’s why the response of these models is comparable with original one. We can reduce this model up to 7th order only because reducing further will lead to deteriorate the performance further as original information of the system will be lost.

Figures 12, 13 shows the variations of $\:\varDelta\:P{g}_{h1}$and $\:\varDelta\:P{g}_{h2}$ when subjected to a disturbance in area 2. The transient and steady state behavior are almost similar; however, differences in peak values and steady state errors are visible in the response patterns.

From the studies, it is seen that the reduced 7th order model is a good approximation of original model as it preserves the important characteristics of the original system. To check the stability of the system, eigenvalues of reduced order models are obtained and shown in Table 2. Eigenvalues of 7th, 8th and 9th order models shows that the system is stable and acceptable at it is.

**Table 2:** Eigenvalues of original & reduced order models using HNA method.

Eigenvalues of original 11th order in closed loop Configuration	Eigenvalues of reduced 9th order model	Eigenvalues of reduced 8th order model	Eigenvalues of reduced 7th order model
-1.2280 + 2.5972i	-3.5963 + 0.0000i	-7.7278 + 0.0000i	-1.0283 + 2.5069i
-1.2280–2.5972i	-1.2236 + 2.5824i	-1.0867 + 2.5284i	-1.0283–2.5069i
-3.4901	-1.2236–2.5824i	-1.0867–2.5284i	-0.8269 + 1.2127i
-1.1019 + 1.4569i	-1.1064 + 1.5099i	-0.9544 + 1.3337i	-0.8269–1.2127i
-1.1019–1.4569i	-1.1064–1.5099i	-0.9544–1.3337i	-0.3828 + 0.4344i
-2.8600	-1.8970 + 0.0000i	-0.3392 + 0.0000i	-0.3828–0.4344i
-2.0709	-0.4780 + 0.3203i	-0.3632 + 0.4159i	-0.2854 + 0.0000i
-1.3134	-0.4780–0.3203i	-0.3632–0.4159i	
-0.5012 + 0.3151i	-0.3945 + 0.0000i		
-0.5012–0.3151i			
-0.3913			

**Fig. 14:**
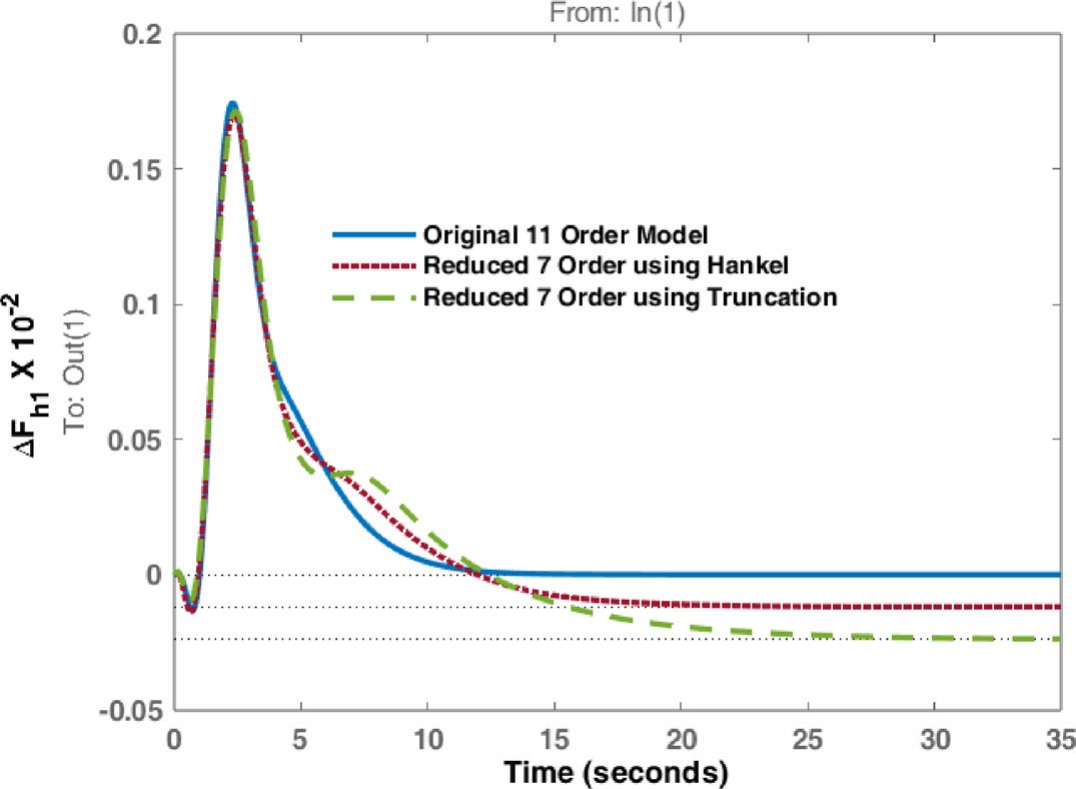
Comparison of dynamic response of change in frequency at area 1 due to disturbance in area 1.

**Fig. 15:**
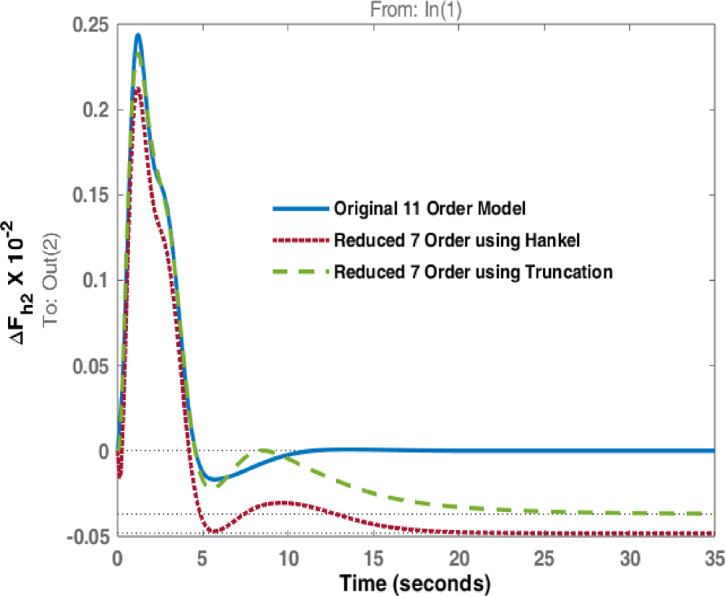
Comparison of dynamic response of change in frequency at area 2 due to disturbance in area 1.

**Fig. 16:**
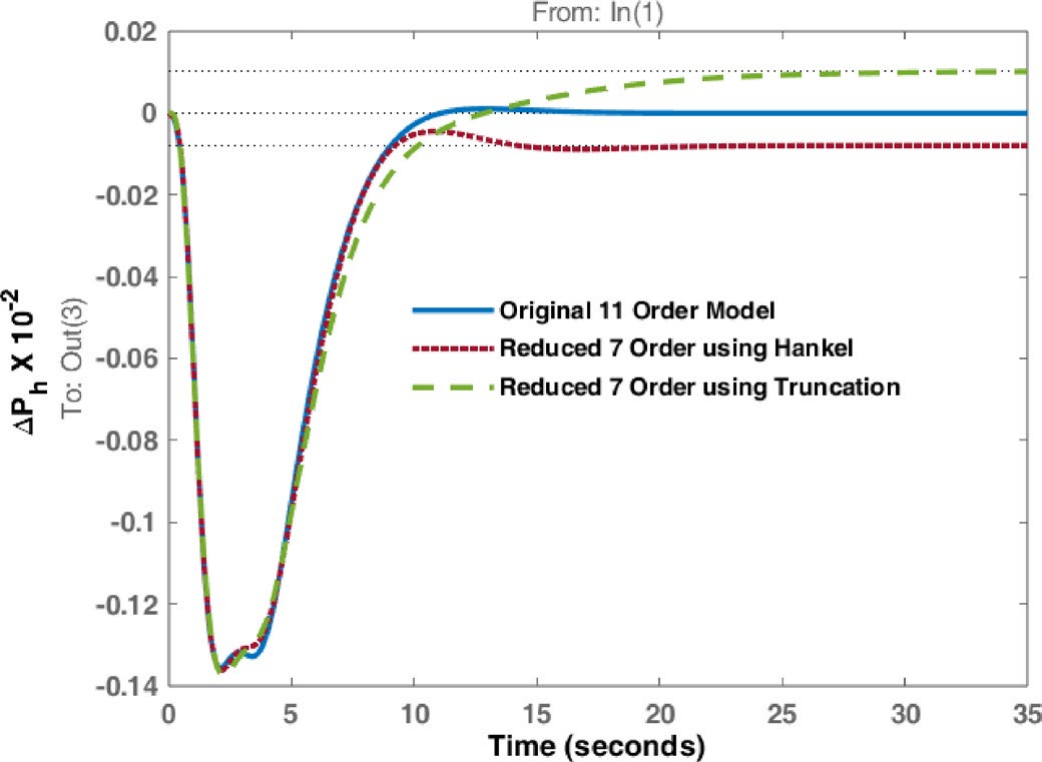
Comparison of dynamic response of $\:\varDelta\:{P}_{h}$ due to disturbance in area 1.

**Fig. 17:**
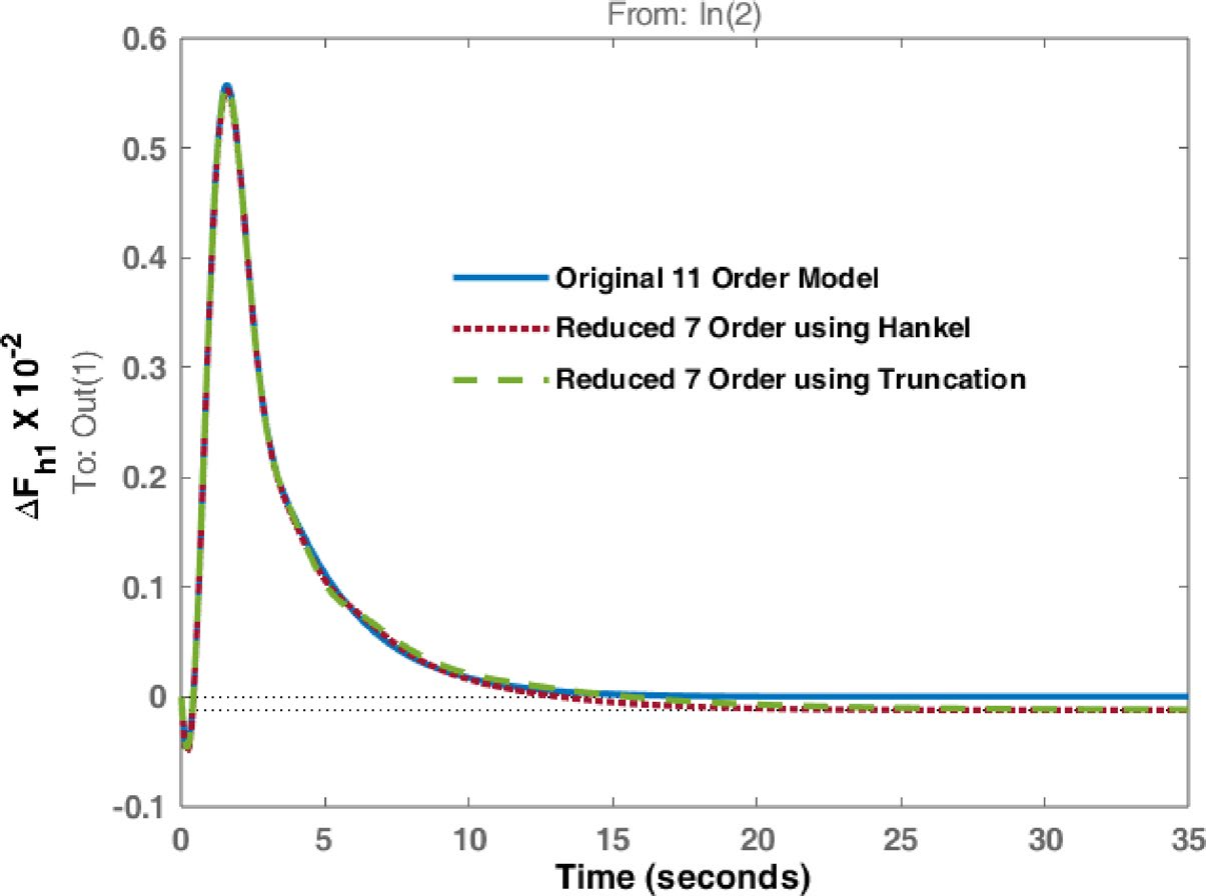
Comparison of dynamic response of change in frequency at area 1 due to disturbance in area 2.

**Fig. 18:**
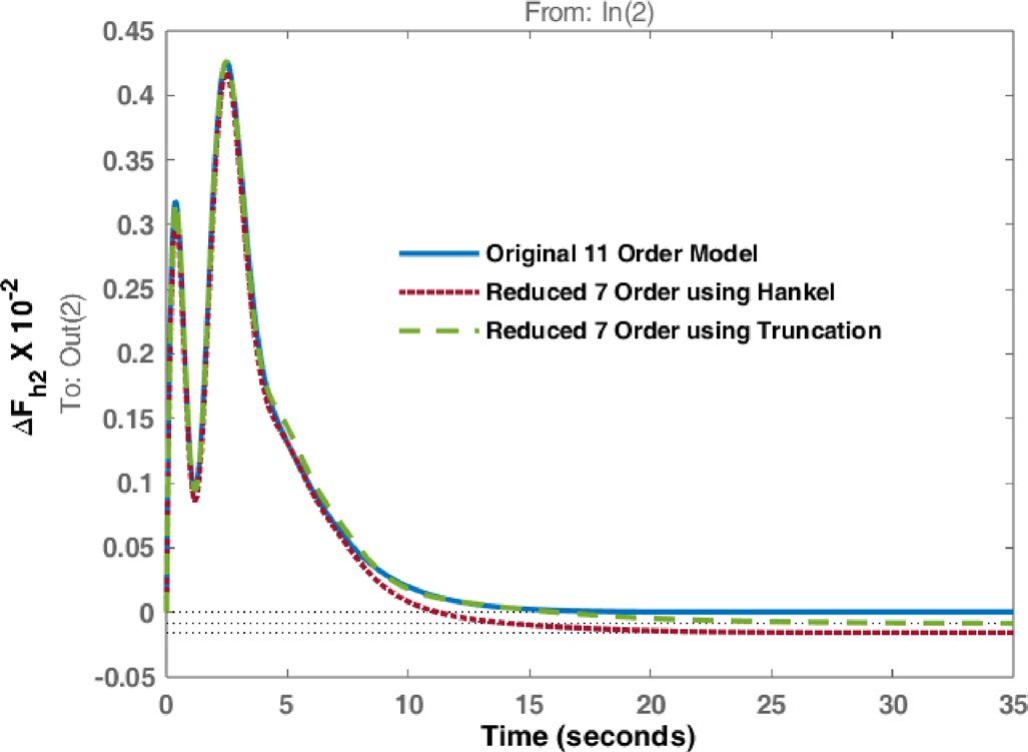
Comparison of dynamic response change in frequency at area 2 due to disturbance in area 2.

**Fig. 19:**
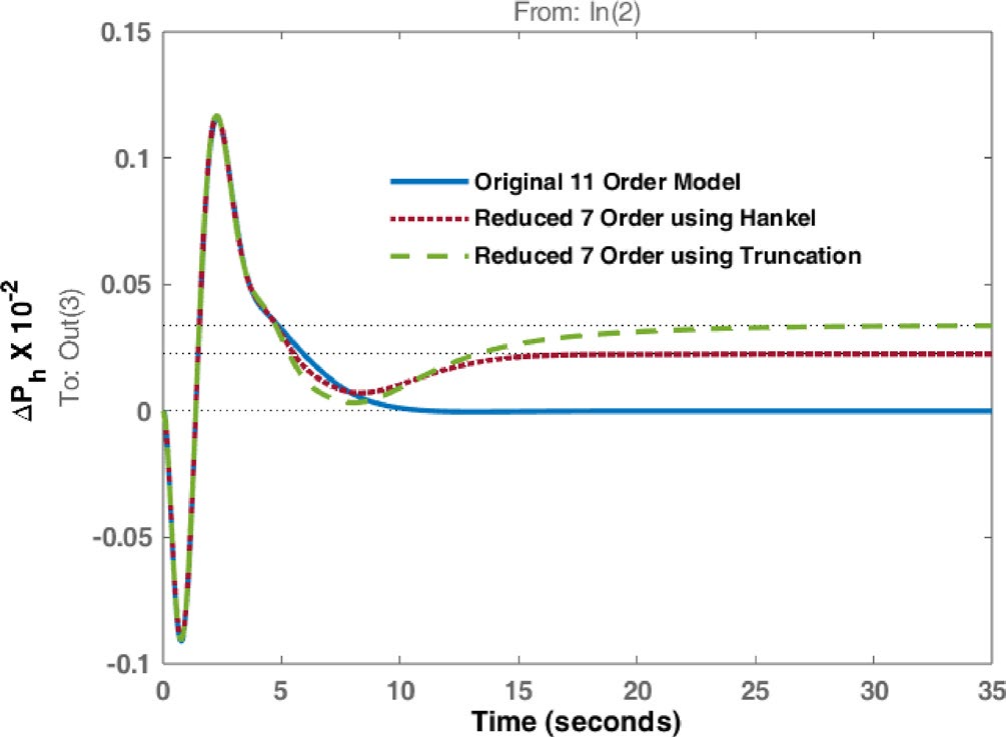
Comparison of dynamic response of $\:\varDelta\:{P}_{h}$due to disturbance in area 2.

The studies are extended with Balanced Truncation method and is applied on the hydro dominated power system in order to match the performance of HNA with Balanced Truncation method. The frequency deviations i.e., $\:\varDelta\:{F}_{h1\:}$& $\:\varDelta\:{F}_{h2\:\:}$and tie line power $\:\varDelta\:{P}_{h\:}$ for the disturbances in area 1 and 2 are observed and investigated. Original system dynamic responses are compared with the reduced order model responses using HNA method and Balanced Truncation method. Figures 14, 15 and 16 represent dynamic behaviour when disturbance occurs at area 1 while Figs. 17, 18 and 19 represent dynamic behaviour when system is subjected to disturbance at area 2. The result shows that the transient response of reduced order model from both methods are comparable but steady state response shows significant deviation. Steady state error is higher in reduced 7th order using Truncation method whereas HNA has less steady state error as compared to balanced truncation.

## Conclusions

This paper introduces the HNA technique to address the AGC problem in hydro dominated power systems. The study validates its findings by analysing a hydropower system interconnected via an AC tie-line, originally modelled as an 11th order system. The research begins with solving the system’s differential equations and subsequently deriving its state-space model.

The HNA method is applied to reduce the system order from 11th to lower orders, aiming to simplify the model and reduce computational time for AGC analysis. The energy states of both the original 11th order and reduced 7th order models are plotted, revealing that states 1 through 7 exhibit high energy levels. This indicates that the system can effectively be reduced to as low as the 7th order. Consequently, the system is approximated to 7th, 8th and 9th order models using HNA method.

The bar chart of the 7th order model demonstrates that high-energy states are preserved while the last four states of the original model can be discarded. Since high-energy states correspond to lower eigenvalues, this reduction retains the essential system dynamics. Additionally, the dynamic responses of the original system and its reduced-order models (7th, 8th and 9th ) are compared to assess AGC performance. The analysis confirms that the 7th order model provides a strong approximation of the original, effectively preserving key AGC characteristics and system response behaviour. It is also observed that all reduce order models are stable as per obtained eigenvalues, however, further reduction may deteriorate the performance and hence not recommended for hydro dominated power system.

In addition, a comparison between the Balanced Truncation method and the HNA method shows that both techniques have the capabilities in showing similar performance, however, HNA outperform in minimizing steady state error from the AGC responses for a reduced order models making it a more effective choice for AGC applications for a higher order and complex systems.

### Future recommendation

The proposed method can be applied to derive a reduced model for more complex having the dynamics of wind or solar PV. This reduced-order model can be utilized for controller design, allowing a comparison of the time required to design controllers for both the reduced and original models. Additionally, it is observed that the model cannot be reduced to an extremely low order. Therefore, the HNA method can be further refined to achieve improved results.

## Supplementary Information

Below is the link to the electronic supplementary material.

Supplementary Material 1

## Data Availability

The data should be available on request from the corresponding author and Gulshan Sharma (gulshans@uj.ac.za ).
