# Supplementary material for: A novel Hankel norm approximation-based AGC for a hydro-dominated power system
Source: Sci Rep. 2026 Jan 16;16:5522. doi: 10.1038/s41598-026-35235-9 (PMC12886902; doi:10.1038/s41598-026-35235-9)
Supplement: Supplementary file 1 — Supplementary Material 1 [file 41598_2026_35235_MOESM1_ESM.docx]

**Appendix [16]**

Description of Symbols:

ΔF_1_, ΔF_2_ = frequency deviation in area-1 and area-2 respectively

ΔP_h_ = change in tie line power

ΔPg_h1_, ΔPg_h2_, =change in governor power in area-1 and area-2 respectively

ΔPt_1_, ΔPt_2_ = change in turbine power in area-1 and area-2 respectively.

$P_{th1}=P_{th2}=rated area Power output=2000MW$,

$T_{gh1}=T_{gh2}=speed governor time constant=5s$,

$$T_{\mathrm{hi}}= Time constant associated with hydro governor of area {'i}^{'} {;T}_{h1}=T_{h2}=48.7s$$

$T_{h3}=T_{h4}=5s$ , $T_{w1}=T_{w2}=hydro turbine time constant=1.0 s$,

$K_{gh1}=K_{gh2}=Speed governor gain=1.0$,

$M_{h1}={Tp_{h1}}/{Kp_{h1}}=\frac{2H_{h1}}{f^{0}}=M_{h2}=Effective rotary inertia=0.167p.u.MW-sqsec$,

$D_{h1}=1/{Kp_{h1}}$=$D_{h2}=Load frequency constant=0.00833 p.u.mW/Hz$,

$R_{h1}=R_{h2}=Speed regulation parameter=2.4 Hz /p.u.MW$,

$$B_{h1}=B_{h2}=frequency bias constant=0.425 p.u.MW/Hz$$

$\Delta P_{dh1}=\Delta P_{dh2}=Incremental change in load demand=0.01 p.u MW$/Hz
